# NDRG1 regulates neutral lipid metabolism in breast cancer cells

**DOI:** 10.1186/s13058-018-0980-4

**Published:** 2018-06-14

**Authors:** Christopher J. Sevinsky, Faiza Khan, Leila Kokabee, Anza Darehshouri, Krishna Rao Maddipati, Douglas S. Conklin

**Affiliations:** 1 Cancer Research Center, Department of Biomedical Sciences, State University of New York, University at Albany, CRC 342, One Discovery Drive, Rensselaer, NY 12144-3456 USA; 2 Electron Microscopy Core Facility, The University of Texas Southwestern Medical Center, 5323 Harry Hines Boulevard, Dallas, TX 75390 USA; 3 Lipidomics Core Facility, Wayne State University, 435 Chemistry Bldg., Detroit, MI 48202 USA

**Keywords:** Marker, Lipogenic, Metabolism, Metabolomics, Aggressiveness

## Abstract

**Background:**

Altered lipid metabolism is an emerging hallmark of aggressive breast cancers. The N-myc downstream regulated gene (NDRG1) gene plays a critical role in peripheral nervous system myelination, as inactivating mutations cause severe demyelinating neuropathy. In breast cancer, elevated NDRG1 expression has been linked to clinical outcomes, but its functional role in breast cancer physiology has remained unclear.

**Methods:**

A meta-analysis of NDRG1 expression in multiple large publicly available genomic databases was conducted. Genome-wide expression correlation and Cox proportional hazards and Kaplan-Meier modeling of clinical outcomes associated with elevated expression were assessed. To study NDRG1 function, gene silencing and overexpression phenotypic studies were carried out in a panel of cell lines representing all major breast cancer molecular subtypes. Changes in cell proliferation, morphology, and neutral lipid accumulation due to altered NDRG1 expression were assessed by high throughput, quantitative microscopy. Comprehensive lipidomics mass spectrometry was applied to characterize global changes in lipid species due to NDRG1 silencing. Labeled fatty acids were used to monitor cellular fatty acid uptake and subcellular distribution under nutrient replete and starvation culture conditions.

**Results:**

NDRG1 overexpression correlated with glycolytic and hypoxia-associated gene expression, and was associated with elevated rates of metastasis and patient mortality. Silencing NDRG1 reduced cell proliferation rates, causing lipid metabolism dysfunction including increased fatty acid incorporation into neutral lipids and lipid droplets. Conversely, NDRG1 expression minimized lipid droplet formation under nutrient replete and starvation conditions.

**Conclusions:**

Here we report that NDRG1 contributes to breast cancer aggressiveness by regulating the fate of lipids in cells that exhibit an altered lipid metabolic phenotype. In line with its role in promoting myelination and its association with altered metabolism in cancer, our findings show that NDRG1 is a critical regulator of lipid fate in breast cancer cells. The association between NDRG1 and poor prognosis in breast cancer suggests it should play a more prominent role in patient risk assessment. The function of NDRG1 in breast cancer lipid metabolism may represent a promising therapeutic approach in the future.

**Electronic supplementary material:**

The online version of this article (10.1186/s13058-018-0980-4) contains supplementary material, which is available to authorized users.

## Background

Elevated N-myc downstream regulated gene (NDRG1) messenger RNA (RNA) and protein expression is found in a subset of many solid tumors, including breast cancer. Autosomal recessive NDRG1 null mutations result in Charcot-Marie-Tooth disease type 4D (CMT4D), a severe demyelinating peripheral neuropathy disorder [1]. CMT4D pathology is thought to be caused by dysfunctional lipid metabolism in Schwann cells, the glia of the peripheral nervous system [2]. However, little functional evidence has been advanced in support of this hypothesis. The NDRG proteins possess an inactive α/β hydrolase fold flanked by intrinsically disordered N and C termini [3], which are subject to extensive post-translational modification. NDRG1 is distinguished from other family members by its transcriptional upregulation in response to stress, including hypoxia, and by its distinctive structural features including a C-terminal metal-binding decapeptide triple repeat that is heavily phosphorylated, and a putative phosphopantetheine attachment site in the core α/β hydrolase domain [4]. The lack of an active site has complicated interpretation of studies exploring NDRG1 function, and insights from studies of NDRG1 in a variety of model systems implicate NDRG1 in several unrelated cellular processes [5–8].

NDRG1 as a prognostic factor in breast cancer remains controversial, as it continues to be cited as both a biomarker of negative prognosis and as a metastasis suppressor [9–13]. Although its function is poorly defined, NDRG1 is a direct transcriptional target of hypoxia inducible factor 1α (Hif1α), Hif2α, and X-box binding protein 1 (XBP1) [12, 14, 15]. NDRG1 protein expression has been associated with high uptake of 18-fluorodeoxyglucose and estrogen receptor (ER)-negative breast cancers in vivo [16], but rather than a role in glycolysis, physical interactors and physiological consequences of NDRG1 malfunction suggest a poorly defined role related to lipid biology in cancer [2, 6, 7, 17]. Like most cancers, aggressive breast cancer subtypes [18] are dependent on elevated glycolytic metabolism [19, 20]. A consequence of the well-characterized dependence on glycolysis of breast cancer cells, de novo lipogenesis is increasingly recognized as a central feature of this metabolism [21–23]. Here, we show that NDRG1 is expressed in a Warburg-like metabolic gene expression program common to many solid tumors, including breast cancer. Several lines of evidence show that NDRG1 performs an important pro-survival function in regulating the fate of lipids in breast cancer cells.

## Methods

### Tissue culture

Cells were purchased from American Type Culture Collection (ATCC) (Manassas, VA, USA): SKBR3 (HTB-30), MCF7 (HTB-22), HCC1569 (CRL-2330), BT474 (HTB20), MDA-MB-231 (CRM-HTB-26), MDA-MB-468 (HTB-132), HEK293T (CRL-3216). Cells were cultured in DMEM/high glucose with L-glutamine, and sodium pyruvate (Hyclone SH30243.01), supplemented with 10% fetal bovine serum (Sigma), in a standard humidified incubator (5% CO_2_), or under hypoxic conditions (1% O_2_). All cells cultured under hypoxia conditions were harvested 24 h after exposure to low oxygen. All cell lines were authenticated in March 2016 by the SUNY-Albany Center for Functional Genomics Molecular Core Facility using a short tandem repeat method (Promega GenePrint 10 system).

### Gene expression manipulation

For lentivirus production, HEK293T cells were transfected using X-treme gene HP transfection reagent (Roche) with Gag/pol, Rev., VsVG (Invitrogen Virapower), and NDRG1 shRNA pLKO.1 plasmids (Dharmacon/GE Healthcare TRC short hairpin RNAs (shRNAs): TRCN0000084043, TRCN0000084044, TRCN0000084045, TRCN0000084046, TRCN0000084047), or negative control plasmids: empty vector (RHS4080) and nontargeting eGFP shRNA (RHS684). Virus infection was performed with 8 μg/ml polybrene (Sigma) and cells selected in medium containing 1 μg/ml puromycin (Sigma). For retrovirus production, ΦNX-Ampho cells [24] were transfected with Flag-tagged versions of NDRG1 cloned in the MarxIV vector [25] and empty MarxIV vector was used as a control. Cells were selected with 200 μg/ml hygromycin B and expression confirmed with anti-Flag antibody (SIGMA mouse M2 antibody produced in mouse). The NDRG1 complementary DNA (cDNA) was obtained in pDSRED N2 [6]. A point mutation (L237P) was found in the NDRG1 coding sequence in this construct and corrected using site-directed mutagenesis before generation of other wild-type and mutant expression plasmids.

### Fluorescence microscopy

For immunofluorescence, fixed cells were permeabilized in PBS + 0.1% Triton X-100 for 15 min at room temperature. All antibodies were diluted in standard antibody diluent: PBS, 0.1% Tween-20, 5% bovine serum albumin. All primary antibody incubations were carried out overnight at 4 °C, followed by three 5-min washes in 200 μl PBS. Secondary antibodies were diluted to 5 μg/ml, and applied to cells for 1 h at room temperature. Cells were then washed once in 200 μl PBS containing 1 μg/ml Hoechst dye (10 min), followed by two consecutive 5-min washes in 200 μl PBS to eliminate unbound antibody and dye, and remaining PBS was aspirated from wells and replaced with fresh PBS for imaging.

Antibodies included mouse anti-FLAG M2 antibody (SIGMA, cat# F1804), rabbit anti-NDRG1 (Cell Signaling Inc., cat# 9485), rabbit anti-pNDRG1 T346 (Cell Signaling Inc., cat# 5482). Rabbit anti-pNDRG1 S330 (Abcam, cat# ab124713), rabbit anti-glyceraldehyde-3-phosphate dehydrogenase (anti-GAPDH) (Cell Signaling Inc., cat# 5174), rabbit anti-cleaved caspase-3 (Asp175) (Cell Signaling Inc., cat #9579) and rabbit anti-phospho-histone H3 S10 (Cell Signaling Inc., cat #9701). Secondary antibodies were from Jackson Immunoresearch and included donkey anti-rabbit IgG (H + L) Cy3 (cat# 711-166-152) and donkey anti-rabbit IgG (H + L) Alexa 647 (cat# 711-606-152).

Images were acquired at × 20 magnification on an inverted fluorescence microscope (Olympus IX-81) fitted with a Retiga 6000 CCD Camera using Metamorph software or on the InCell Analyzer 2200 high content microscopy platform (GE Healthcare). All images were acquired using the same exposure time to allow quantitative analysis and comparison of staining intensity. For cells imaged using the IN Cell Analyzer 2200 (GE Healthcare), a minimum of four images per well from a minimum of three wells were collected for each condition, and data were summarized at the well-level for statistical comparisons. Exposure times were set to allow quantitative analysis of intensity using statistical tests comparing the brightness of objects (e.g., lipid droplets). Images were analyzed using GE Healthcare In Cell Analyzer software granule and nuclei counting algorithms. For lipid droplet granules, sensitivity was set to the most stringent threshold to reduce nonspecific granule counting. Particle size was set to the range 0.1–2 μm^2^. Nuclei were also counted - minimum size 40 μm^2^. For the analysis of pNDRG1 puncta dynamics in response to endoplasmic reticulum stress, 5–250 pixel foci with circularity of 0.25–1.0 were segmented using the triangle algorithm, and divided by the total number of nuclei in each image to express puncta per cell.

For live cell imaging, cells transduced with green fluorescent protein (GFP) and NDRG1 targeting virus were plated in 6-well plates, allowed to attach for 24 h, refed, and monitored by phase contrast microscopy for 48 h. Live cell microscopy was conducted using the Invitrogen EVOS FL Auto Cell Imaging System with a pre-warmed, humidified, and 5% CO_2_ equilibrated incubation chamber. Images were acquired at 10-min intervals with autofocus on.

### Transmission electron microscopy

Hs578T cells expressing three different shNAs targeting NDRG1 and one targeting enhanced (e)GFP were collected at one week post selection. Cells were grown in 10-cm dishes to 80–90% confluence and fixed in 0.1 M cacodylate pH 7.4, 2.5% glutaraldehyde (Electron Microscopy Sciences). Cells were fixed at 37 °C for 15 min, followed by scraping of cells, collection by centrifugation at × 100 g, and resuspension in 10 ml fresh fixation buffer and storage at 4 °C. After three rinses with 0.1 M sodium cacodylate buffer, cell pellets were embedded in 3% agarose and sliced into small blocks (1mm^3^), rinsed with the same buffer three times and post-fixed with 1% osmium tetroxide and 0.8% potassium ferricyanide in 0.1 M sodium cacodylate buffer for 1.5 h at room temperature. Cells were rinsed with water and stained en bloc with 4% uranyl acetate in 50% ethanol for 2 h. Cells were dehydrated with increasing concentration of ethanol, transitioned into propylene oxide, infiltrated with Embed-812 resin and polymerized in an oven at 60 °C overnight. Blocks were sectioned with a diamond knife (Diatome) on a Leica Ultracut 6 ultramicrotome (Leica Microsystems) and collected onto copper grids, post-stained with 2% aqueous uranyl acetate and lead citrate. Images were acquired on a Tecnai G2 spirit transmission electron microscope (FEI) equipped with a LaB6 source using a voltage of 120 kV.

### Lipidomics mass spectrometry

SKBR3 cells transduced with shRNAs targeting NDRG1 or eGFP were grown to 80%–90% confluence and harvested by trypsinization at 14 days post selection (including 7 days in culture without puromycin). As soon as cells lifted, they were immediately suspended in ice cold medium and kept on ice while a small fraction was counted. Cells were then divided into 1 or 2 million cell aliquots (shotgun versus targeted analysis, respectively), rinsed twice in ice cold PBS by low-speed centrifugation at 4 °C and wash was aspirated, and pellets frozen in a dry ice/ethanol bath and stored at − 80 °C until analysis. Silencing levels and lipid droplet quantities were determined in a sample of cells grown in microtiter plates as described above, by anti-NDRG1 immunofluorescence and boron-dipyrromethene (BODIPY) staining, respectively.

Lipid classes were analyzed by the multiple precursor ion scanning (MPIS) method for triacylglycerides, diacylglycerides, phophatidylcholines, phosphatidylethanolamines, phsophatidylserines, phosphatidylglycerols, phosphatidic acids, and phosphatidylinositols following published protocols [26, 27]. Monoacylglycerols, cholesterol esters, sphingomyelins, and ceramides were analyzed by LC-MS methods using multiple reaction monitoring (MRM) methods [28, 29]. To generate a heatmap for visualization of various lipid levels, data were first standardized by normalizing to row means as a fold difference and species not detected in all samples, and extreme outliers were removed (*n* = 142 species remaining). The online cluster analysis tool CIMiner was used to generate heatmaps using Euclidian distance and colors were represented using quantiles (https://discover.nci.nih.gov/cimminer/home.do).

Targeted LC MS/MS analysis of cholesterol esters was from five biological replicates for each condition, and represented as ng lipid/million cells. Targeted LC MS/MS analysis of triglycerides was from six biological replicates per condition, and represented as micrograms of lipid/micrograms of protein. Individual species were compared using the two-sided Student’s *t* test. Each lipid class (e.g., cholesterol esters, triacylglycerol) was also analyzed as an aggregate of all individual species detected and also compared using the two-sided Student’s *t* test.

### mRNA expression analysis

The breast-cancer-specific mRNA expression characteristics were examined for genes coexpresssed or anti-correlated with NDRG1 expression in order to better understand relationships between NDRG1 and markers reflecting intrinsic molecular subtypes. The cBio portal was accessed in order to analyze global gene expression patterns across 18 human solid tumor types [30]. The online tool KM plotter was used to establish query criteria and generate KM plots, hazard ratios, 95% confidence intervals, and *p* values [31]. Additional cohorts were analyzed by accessing independent patient cohorts with the online tool SurExpress (http://bioinformatica.mty.itesm.mx:8080/Biomatec/SurvivaX.jsp). NDRG1, NDRG1 + MYC, or the 42-member NDRG1-associated gene signature was queried for relationships with adverse outcomes (metastasis-free survival, or recurrence-free survival). The mRNA expression of *NDRG1* in > 1000 cell lines was downloaded from the Broad Institute CCLE portal: https://portals.broadinstitute.org/ccle/home. Breast cancer cell lines were filtered, ranked according to expression level, and plotted to evaluate the range of expression in characterized cell lines. Cell lines chosen for in vitro studies are indicated.

Microarray analysis of SKBR3 cells expressing NDRG1 shRNA1 or vector control were performed with biotin-labeled cDNA from three independent biological replicates hybridized over 16 h to Affymetrix Gene 2.0 ST arrays and scanned on an Affymetrix Scanner 3000 7G using AGCC software. The resulting CEL files were analyzed for quality using Affymetrix Expression Console software and were imported into GeneSpring GX11.5 (Agilent Technologies) where the data were quantile normalized using PLIER and baseline transformed to the median of the control samples. Data sets are available at GEO, Accession number: GSE112841.

### Fatty acid conjugated BODIPY tracer experiments

The sixteen carbon BODIPY FL C16 (Invitrogen, cat# D3821) was used to study fatty acid uptake and intracellular distribution in stable shNDRG1 and shGFP expressing SKBR3 cells (cultured as in lipidomics experiments described above). A 2 mM stock solution was prepared in culture medium supplemented with 0.1% fatty acid free bovine serum albumin (Goldbio cat# A-421-100), and diluted in DMEM/high glucose with L-glutamine, and sodium pyruvate + 10% FBS to achieve the desired concentrations. Cells were seeded at 10,000 viable cells per well, allowed them to adhere for 24 h, and medium was replaced with C16-BODIPY at 100 μM, 50 μM, 25 μM and 12.5 μM in complete DMEM and cells were cultured under normal conditions for 16 h in the presence of the tracer. After 16 h, excess tracer was removed by three consecutive washes in complete DMEM, followed by the fixation protocol described above.

To quantify total tracer uptake, an image analysis routine was established to measure the intensity of cell-sized objects above a background threshold. Cell nuclei were counted, and average C-16 BODIPY signal was expressed as the ratio of overall signal divided by the number of nuclei in each field of view. To quantify lipid-droplet-specific signal, the granule counting and measurement algorithm described above was used. The ratio of lipid-droplet-specific C-16 BODIPY signal to total C-16 BODIPY signal was computed to reflect the flow of tracer fatty acid to lipid droplets.

In MCF7 cells, a protocol to induce lipid droplet formation and live cell tracer incorporation through starvation in Hanks buffered saline solution (HBSS) was developed based on published methods [32]. HBSS consists of 0.137 M NaCl, 5.4 mM KCl, 0.25 mM Na2HPO4, 0.63 mM glucose, 0.44 mM KH2PO4, 1.3 mM CaCl2, 1.0 mM MgSO4, and 4.2 mM NaHCO3. Stable MCF7 cell lines transduced with retroviral full length NDRG1–3X FLAG and retroviral empty vector were plated as described above cultured in ether complete medium or HBSS. For live cell experiments, BODIPY 558/568 C12 (C12-BODIPY) was added to initial culture medium at 100 μM to allow cellular uptake, and followed by extended chase periods. Samples were fixed and stained for lipid droplets as described above. To elaborate the time dependence of the phenomenon and rule out tracer specific effects, lipid droplet formation by cells grown without C12-BODIPY tracer were monitored over the course of 4 days by staining with BODIPY 493/503 using analysis methods described above.

## Results

### NDRG1 is a biomarker of aggressive breast cancers

**Fig. 1:**
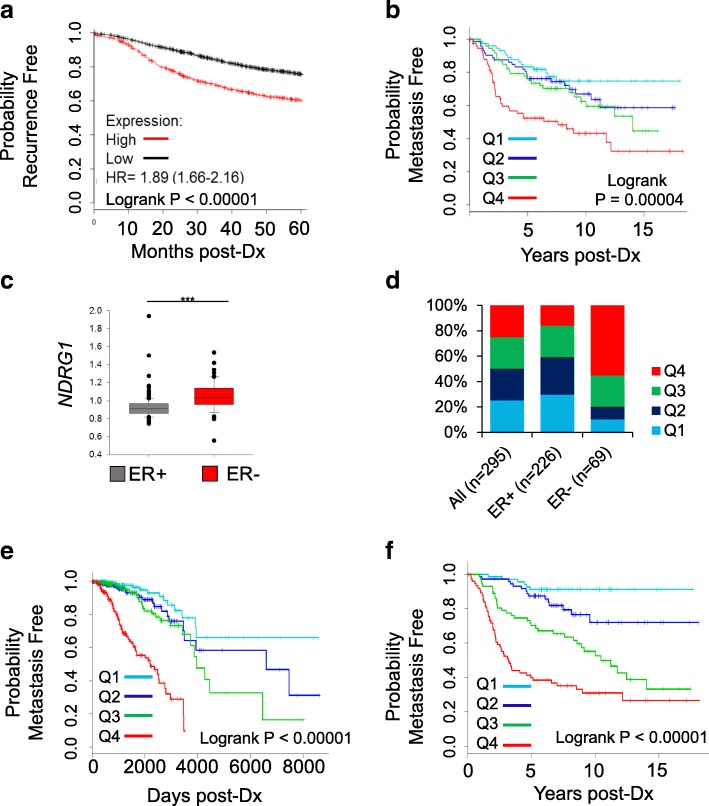
*NDRG1* expression is associated with adverse outcomes and altered metabolism in breast cancer. **a** Meta-analysis of 23 distinct breast cancer cohorts (*n* = 3554 subjects). Kaplan-Meier curve of recurrence-free survival analysis of *NDRG1* mRNA expression (upper quartile (Q) versus lower three quartiles). Post-DX, Post-diagnosis. **b** Metastasis-free survival of *NDRG1* quartiles in the Van’t Veer cohort (*n* = 295). **c** *NDRG1* expression in estrogen receptor (ER)+/− groups of the the Van’t Veer cohort. **d** The distribution of total Van’t Veer cohort NDRG1 expression quartiles in ER+ and ER- tumors. The unequal distribution further reflects the data in (**c**). **e**, **f** Metastasis-free survival using the 42-gene signature in The Cancer Genome Atlas breast cancer cohort (*n* = 962), and the Van’t Veer breast cancer cohort (n = 295)

Previous studies of NDRG1 expression in cancer have been performed on cohorts of limited sizes, and have led to the conclusion that NDRG1 is a metastasis suppressor [9–11, 33]. However, several lines of evidence contradict NDRG1 as a suppressor of metastasis [12, 13]. So that we might resolve questions on the link between NDRG1 expression and disease outcome, we examined large, recently established, public repositories of clinical breast cancer data. A large meta-analysis was conducted examining the association between NDRG1 expression and recurrence-free survival in 23 publicly available breast cancer mRNA expression data sets representing 3554 subjects with breast cancer assessed as a single aggregated cohort [31]. Because *NDRG1* copy number or mRNA expression is altered in approximately 25% of all breast cancers in the The Cancer Genome Atlas (TCGA) data set (Additional file 1: Figure S1), patients in the upper quartile of NDRG1 mRNA expression were compared with patients in the lower three quartiles. Subjects expressing high *NDRG1* exhibit an almost twofold higher risk of disease recurrence 5 years after diagnosis in this aggregate cohort (Fig. 1a). The 23 individual cohorts were also analyzed individually to assess potential bias: 19/23 cohorts show that high *NDRG1* is associated with decreased time to recurrence, 3 show no separation of Kaplan-Meier curves, and a small cohort (*n* = 77 subjects) exhibits an association between high *NDRG1* and positive outcome (Additional file 1: Figure S2).

An independent cohort of 295 patients in whom metastasis was tracked was examined to evaluate the relationship between metastasis and *NDRG1* expression in more detail [34]. Again, *NDRG1* mRNA expression level stratified patients into poor and favorable prognostic subsets (Fig. 1b). Importantly, elevated NDRG1 expression is a more consistent biomarker of negative prognosis than the genomically co-amplified *MYC* oncogene, ruling out a simple confluence of high *NDRG1* expression with *MYC* amplification as a driver of poor prognosis (Additional file 1: Figure S3A-C). This meta-analysis significantly strengthens the link between elevated *NDRG1* expression and poor prognosis in breast cancer.

### NDRG1 is correlated with an aggressive metabolic gene expression profile in breast cancer and other solid tumor types

Genome-wide *NDRG1* coexpression patterns were assessed to investigate links between expression levels and recurrent genetic alterations and previously described intrinsic molecular subtypes were present. Pairwise mRNA expression-level correlation between NDRG1 and all other genes analyzed in the TCGA data set were examined. The estrogen receptor (*ESR1*) is near the top of the list of negatively correlated genes. Other negatively correlated genes include well-established markers of luminal breast cancer: *GATA3*, *FOXA1*, and *PGR* (Additional file 2: Table S1). Positive correlation was common between *NDRG1* and genes associated with angiogenesis, glycolysis, hypoxia, and basal cell lineage, illustrating that high *NDRG1* expression is more common in ER^−^ breast cancers (Fig. 1c, d, Additional file 2: Table S2). Previous work has shown *NDRG1* expression to be part of an angiogenesis-related gene signature associated with metastasis in breast cancer [12]. These correlations indicate that *NDRG1* expression is strongly associated with breast tumors with high glycolytic and angiogenic gene expression.

The TCGA breast cancer analysis included quantification of the Thr346 phosphorylated form of NDRG1, but not the total protein, by reverse phase protein array [18]. Proteins negatively correlated with phospho-NDRG1 mirrored the results of the mRNA associations (Additional file 2: Table S3), as did an analysis of mRNA levels in *NDRG1* altered and unaltered cases (Additional file 2: Table S4), corroborating a link between NDRG1 and aggressive forms of breast cancer.

Analysis of *NDRG1* mRNA correlation was extended to include 18 solid tumor types analyzed by the TCGA (Data set S1) to reveal common elements of an underlying biological process, illuminating the physiological action of *NDRG1*. In each cancer type, genes with mRNA with a Pearson’s product moment correlation coefficient (*R*) ≥ 0.40 were identified as NDRG1-associated genes: 41 genes with expression that was correlated with *NDRG1* in at least six individual data sets (≥ 33%) were found to encode nine of ten common glycolysis pathway enzymes, glycolysis regulatory proteins, and downstream regulators of tricarboxylic acid cycle (TCA) cycle entry; regulators of angiogenesis; and several oxidoreductases implicated in oxidative protein folding and signaling under endoplasmic reticulum stress and hypoxic conditions (Additional file 2: Table S5). This pan-cancer correlation with hallmarks of metabolic adaptations underscores the link between NDRG1 and altered cancer metabolism, and the gene list is a potent prognostic signature in breast cancer and several other solid tumors (Fig. 1e, f, and data not shown).

### NDRG1 expression is highly variable in breast cancer cell lines and displays distinct subcellular localization

**Fig. 2:**
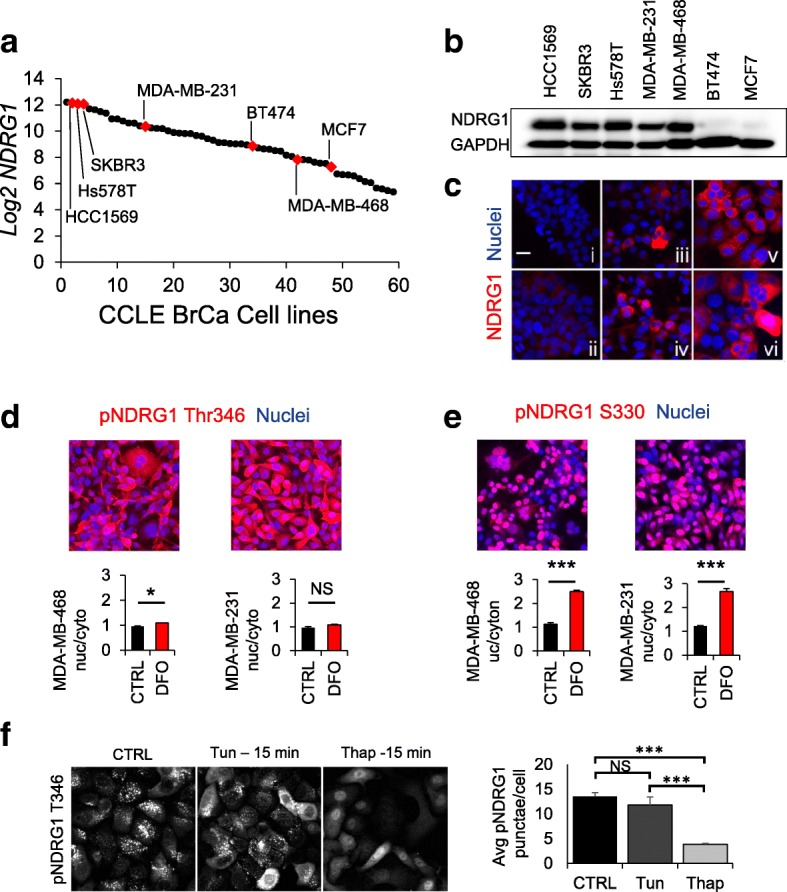
NDRG1 expression level and localization variability in breast cancer cell lines. **a** Cancer Cell Line Encyclopedia (CCLE)-analyzed breast cancer cell lines ordered by *NDRG1* expression level - cell lines selected for in vitro analysis are indicated. **b** Immunoblots of NDRG1 and GAPDH loading control from crude lysates (10 μg total protein/lane). **c** Immunofluorescence analysis of NDRG1 in breast cancer cell lines, scale = 25 μm: **i** MCF7, **ii** BT474, **iii** MDA-MB-231, **iv** MDA-MB-468, **v** SKBR3, **vi** HCC1569. **d, e** NDRG1 pT346 and pS330 immunofluorescence and localization in MDA-MB-231 and MDA-MB-468 cells treated with 100 μM deferoxamine (DFO): nucleus:cytoplasm ratio is shown: *N* = 3/group. **f** pNDRG1 T346 immunofluorescence localization to puncta in SKBR3 cells: thapsigargin (Thap) (1 μM) but not tunicamycin (Tun) treatment (5 μg/ml) disperses puncta. Data are represented as mean ratio +/− SD. *p < 0.001, ***p < 1 × 10^− 10^. CRTL, control; NS, not significant

Since the role of NDRG1 in altered cancer metabolism is poorly characterized, in vitro studies of NDRG1 were carried out in a range of breast cancer cell lines to assess its function. Expression of NDRG1 in vivo is dynamic and dependent on microenvironment cues such as hypoxia or iron limitation [12, 14, 15]; nevertheless, in vitro studies represent the most direct approach to examine protein function in cancer cells. *NDRG1* mRNA expression in the 59 breast cell lines characterized by the Cancer Cell Line Encyclopedia (CCLE) was assessed [35]. Expression levels varied widely, and seven cell lines were selected with a range of *NDRG1* mRNA expression spanning 17-fold (Fig. 2a). The cell lines also represent the full spectrum of major intrinsic molecular subtypes of breast cancer (Additional file 2: Table S6). A wide range of NDRG1 protein expression was also verified. Immunoblots show that HCC1569 expresses the highest level of NDRG1 protein, followed by Hs578T, SKBR3, MDA-MB-231, MDA-MB468, BT474, and MCF7 (Fig. 2b). Similar trends are apparent in immunofluorescence analysis of fixed cells and immunoblots of phosphorylated proteins (Fig. 2c and Additional file 1: Figure S4A). The observed expression profiles are mostly consistent with mRNA levels from the CCLE, and also agree with the preponderance of evidence linking high levels of NDRG1 expression with ER-negative tumors (Fig. 1c, d and Additional file 2: Tables S1–S4).

In addition to total protein levels, we noted differential localization of two phosphorylated forms of NDRG1. The C-terminus of NDRG1 has been shown to be phosphorylated on at least 29 residues [36, 37], but differential phosphorylation states linked with divergent localization or function have not been reported. The total protein, NDRG1 phospho-Thr346 (unique to NDRG1 decapeptide triple repeat), and NDRG1 phospho-Ser330 (conserved in all NDRG proteins) were probed by immunofluorescence microscopy. Immunofluorescence analysis of NDRG1 phospho-Ser330 exhibits an even cellular distribution. NDRG1 phospho-Thr346 in SKBR3 cells showed punctate cytoplasmic patterns consistent with localization to an organelle/endosome/vesicle, suggesting at least partial exclusivity with the Ser330 phosphorylated pool. Remarkably, in addition to upregulation of the total protein, pNDRG1 Ser330 redistributed almost entirely to the nucleus when iron was deprived by chelation with deferoxamine (DFO), which mimics hypoxia, while NDRG1 phospho-Thr346 remained cytoplasmic (Fig. 2d, e and Additional file 1: Figure S4B-D). Because NDRG1 has been implicated in endosomal trafficking, tunicamycin and thapsigargin were used to assess the sensitivity of these structures to inhibition of the endo-lysosomal system [6, 38]. A brief thapsigargin treatment dispersed puncta completely, while tunicamycin had no effect in the acute setting (Fig. 2f). These differences between modified forms of NDRG1 show that distinct pools are sensitive to distinct stimuli, which may explain some of the divergent and apparently unrelated functions attributed to NDRG1 [39].

### NDRG1 expression is critical to breast cancer cell proliferation, viability, and morphology

**Fig. 3:**
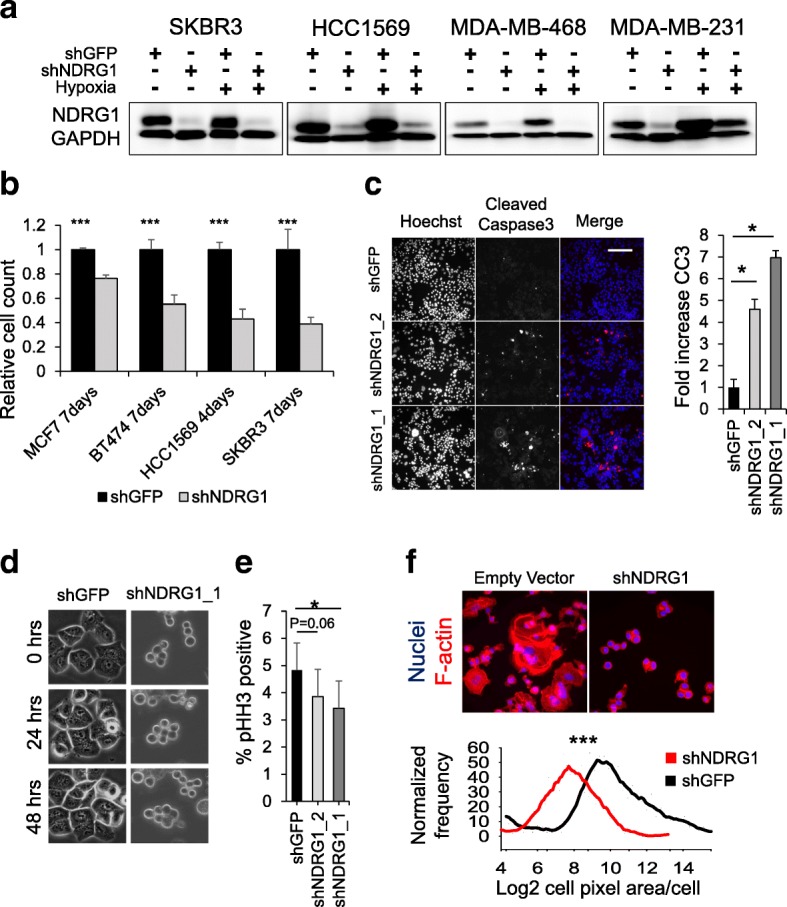
NDRG1 is required for normal breast cancer cell proliferation, viability and morphology. **a** Immunoblots of NDRG1 and GAPDH loading control after selection of four stable lentiviral transduced shRNA expressing cell lines. A green fluorescent protein (GFP) targeting shRNA serves as negative control, and silencing was verified at 1% O_2_. **b** Cell proliferation assays: cell number was compared by comparing NDRG1 silenced cell numbers to control at the times indicated: ***p < 0.0001, *N* = 3/group - see Additional file 1: Figure S5. **c** Cleaved caspase 3 (CC3) immunofluorescence analysis of stable shRNA expressing SKBR3 cells at 1 week post selection. CC3 positive area was normalized to total cell area and each population compared: shNDRG1_1 *p* = 2.5 × 10^− 6^, shNDRG1_2 *p* = 1.2 × 10^− 4^, scale = 100 μm, *N* = 12/group. **d** Individual frames from a 48 h live cell microscopy analysis of SKBR3 cells expressing the indicated shRNAs: *N* = 3/group, see Additional files 3, 4, 5, 6, 7 and 8: Movies S1–S6. **e** Mitotic cell fractions comparing the percentage of histone H3 phsopho-serine10 positive cells in the indicated shRNA expressing cell lines: shNDRG1_1 *p* = 0.02, shNDRG1_2 *p* = 0.06, *N* = 12/group. **f** Comparison of individual cell two-dimensional area based on phalloidin stain (*p* < 0.001, Mann-Whitney U test, *N* = > 650 cells each group). Log2 two-dimensional area is shown. All bar graphs represent means +/− SD. Comparisons were by two-sided Student’s *t* test unless indicated otherwise

To study NDRG1 function in breast cancer cells, lentiviral shRNAs targeting NDRG1 and corresponding GFP targeting controls were used to study the effect of reducing NDRG1 protein levels by silencing its expression (Fig. 3a and Additional file 1: Figure S4A). Beginning at 5 days post-selection, proliferation rates of NDRG1 silenced and control shRNA cells were monitored by comparing nuclei counts at different time points with initial population sizes. SKBR3, MCF7 and BT474 were monitored at 7 days and HCC1569 at 4 days after plating (Fig. 3b), and a series of SKBR3, MDA-MB-231, and MDA-MB-468 time points were also monitored over 7–9 days (Additional file 1: Figure S4E-G). An NDRG1 silencing-dependent reduction in cell proliferation was observed in each of the six cell lines. The magnitude of reduced proliferation was variable, but roughly followed endogenous expression levels, with the most modest effects observed in MCF7, and the greatest effects seen in SKBR3, HCC1569, and MDA-MB-231.

Markers of apoptosis and mitosis were assayed by immunofluorescence microscopy to determine the mechanism of reduced cell proliferation. Silencing NDRG1 in SKBR3 cells increases the cleaved caspase 3 immunofluorescence positive area fraction by 4.5–7-fold (Fig. 3c). This indicates that NDRG1 contributes to cell survival in SKBR3 cells, but the actual fraction of apoptotic cells is small, and NDRG1-depleted cells continue to proliferate. We also analyzed SKBR3 mitotic cell fractions in fixed cells and monitored cell division rates by live cell microscopy. Live cell microscopy showed that NDRG1 depleted SKBR3 cells exhibit a twofold reduction in cell division rates. In addition, a significant morphology change in NDRG1-depleted SKBR3 cells is observed (Fig. 3d, Additional files 3, 4, 5, 6, 7 and 8: Movies S1–S6). Analysis of the mitotic cell marker Histone H3 phospho-Ser10 showed that NDRG1 depletion results in a significant reduction in the mitotic cell population size with one shRNA and a trend toward reduction with a second shRNA (Fig. 3e). We also noted that NDRG1-depleted SKBR3 cells appear smaller, and retain a round morphology throughout the cell cycle, while control hairpin-expressing cells undergo repetitive cycles of post-mitosis flattening, followed by rounding upon entry into mitosis (Fig. 3d and Additional files 3, 4, 5, 6, 7 and 8: Movies S1–S6). In fixed F-actin and nuclear counterstained cells, analysis of single cell populations showed that NDRG1 depletion results in approximately twofold reduction in median F-actin area/cell relative to control (Fig. 3f). Thus, NDRG1 expression is essential to the maintenance of cell proliferation rates, viability, and morphology in breast cancer cells, especially in cell lines with high expression levels.

### NDRG1 affects lipid metabolism in breast cancer cells

**Fig. 4:**
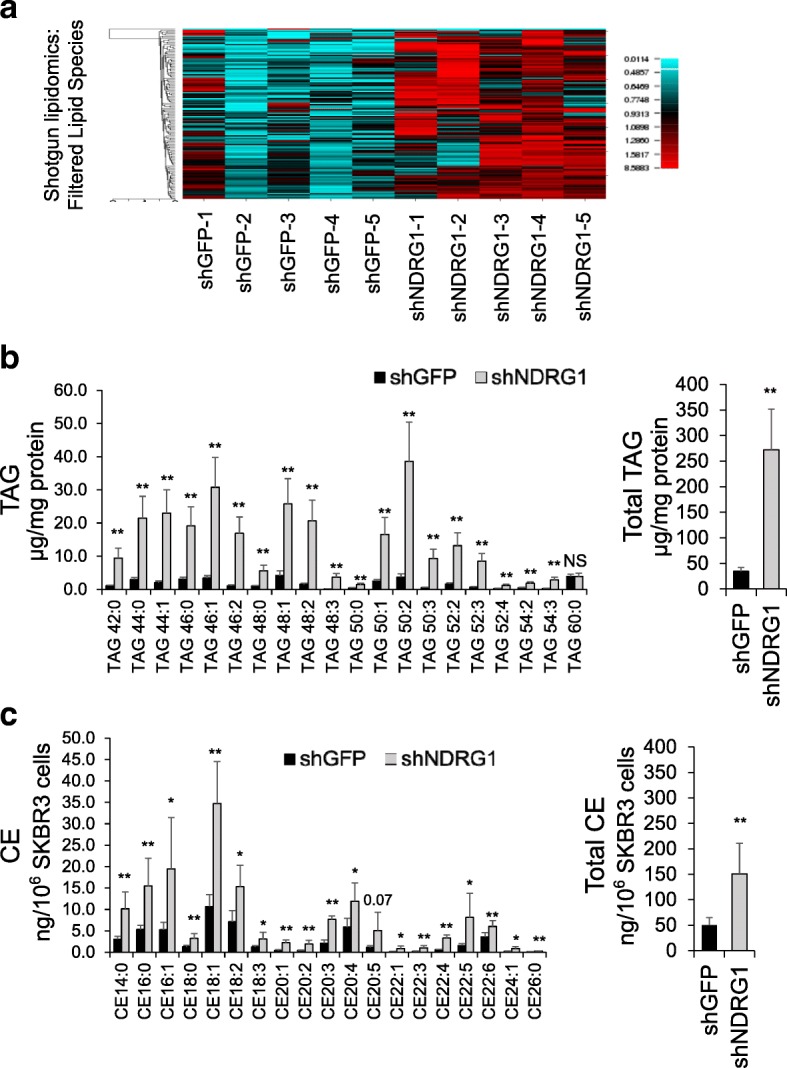
NDRG1 depletion causes increased levels of major lipid species. **a** Semi-quantitative shotgun LC MS/MS of major lipid species in SKBR3 cells. Heatmap represents fold differences in species detected in all samples standardized relative to row means. Color range 0.01–8-fold relative to row mean, *N* = 5 per group. See Additional file 1: Figure S5 for quantification of an independent analysis (**b**) in micrograms of triacylglycerol/mg protein. Individual species and sums are plotted separately (**c**) in nanograms of cholesterol ester per million cells. Sums of individual species and cholesterol ester (CE) species are plotted separately: *N* = 6 for TAG and *N* = 5 for CE analysis. Data represent mean +/− SD, comparisons were by the two-sided *t* test: **p < 0.01, ***p < 0.00001. TAG, triacylglycerols; GFP, green fluorescent protein

The demyelinating peripheral neuropathy Charcot Marie Tooth disease type 4D (CMT4D) is caused by homozygous null mutations in the NDRG1 gene [1]. Since myelination is a lipid metabolism-intensive and trafficking-intensive process, we investigated the impact of decreased NDRG1 levels on lipid metabolism in SKBR3 cells by shotgun mass spectrometry lipidomics. Quantities of several diverse lipid species including structural, storage, biosynthetic intermediates and signaling molecules are increased due to NDRG1 depletion, whereas very few lipids are reduced due to silencing (Fig. 4a and Additional file 1: Figure S5). Consistent with the lack of a catalytic active site, changes in individual species reveal no obvious links between NDRG1 silencing and the inhibition or acceleration of a single biochemical reaction. Analysis of changes in aggregate lipid species performed by comparing the sums of each lipid class between cell lines show the largest effects on the two main storage lipids in NDRG1 silenced cells, triacylglycerols (TAG) and cholesterol esters (CE), with smaller but significant increases in other species (Fig. 4a and Additional file 1: Figure S5).

TAGs and CEs were analyzed by quantitative targeted LC MS/MS, extending the depth of species analyzed within each class of lipid. In accordance with the shotgun analysis, overall TAG abundance increased 7.8-fold, and nearly every species showed a significant increase in the NDRG1-depleted cells (Fig. 4b). Aggregate CE levels increased threefold, and each individual species exhibited increased abundance as well (Fig. 4c). This indicates that NDRG1 regulates neutral lipid storage in SKBR3 cells and that NDRG1-dependent lipid homeostasis in breast cancer cells is important for optimal cell viability.

### NDRG1 depletion results in increased lipid droplet formation in breast cancer cells

As neutral lipids are primarily stored in lipid droplets, quantitative high-content microscopy analysis was applied to characterize the relationship between NDRG1 expression and lipid droplet formation in several additional cell lines. Lipid droplets are composed of a neutral lipid core of mainly TAGs and/or CEs surrounded by a protein and phospholipid monolayer [40]. The fluorescent neutral lipid stain BODIPY 493/503 was used together with an automated granule segmentation and quantification image analysis algorithm to characterize neutral lipid foci in NDRG1 silenced cells [41].

**Fig. 5:**
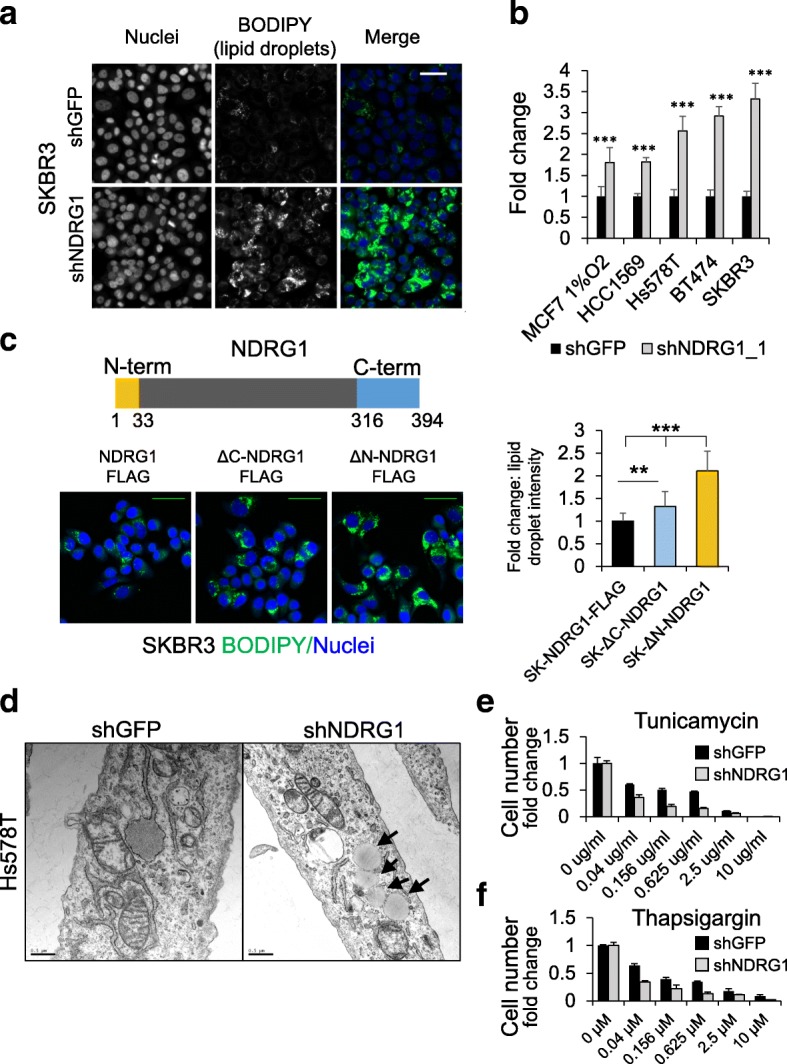
NDRG1 depletion increases neutral lipid storage in lipid droplets. **a** Boron-dipyrromethene (BODIPY) 493/503 and Hoechst-stained stable SKBR3 cells expressing NDRG1 or green fluorescent protein (GFP) targeting shRNA at 2 weeks post selection. Scale bar = 25 μm. **b** Comparison of BODIPY-positive lipid droplet specific signal/cell in stable SKBR3, MCF7 1% O_2_, MDA-MB-231, MDA-MB-468, and HCC1569 expressing the indicated shRNAs: all *N* = 6 and ***p < 0.00001. See Additional file 1: Figure S6 for related data. **c** Comparison of lipid droplet signal in stable SKBR3 cells expressing FLAG tagged full-length NDRG1 and N and C terminal truncations as indicated. *N* = 12, **p < 0.01, ***p < 0.00000001, scale bar = 25 μm. **d** Electron microscopy analysis of Hs578T cells +/− NDRG1 depletion. Arrows indicate lipid droplets. Scale bar = 0.5 μm. **e**-**f** Analysis of thapsigargin and tunicamycin and survival by counting Hoechst-stained nuclei 48 h post stable SKBR3 +/− NDRG1 depletion. Plots represent surviving cell fraction compared to untreated cells, *N* = 3. All bar graphs represent mean +/− SD and comparisons were by the two-sided *t* test. Scale bars = 25 μm

Image analysis results from the BODIPY lipid droplet assay consistently showed that NDRG1 depletion causes increases in lipid droplet formation and intensity relative to control shRNA in all cell lines examined. This effect was largest in SKBR3 cells, where NDRG1 depletion caused a 3.3-fold increase in lipid droplet staining intensity (Fig. 5a, b). NDRG1-depleted HCC1569, Hs578T, and BT474 cell lines also exhibited a significant increase in lipid droplet staining intensity, with 1.8, 2.6, and 2.9-fold increases relative to control, respectively (Fig. 5b). Microarray analysis of transcriptional changes in NDRG1-depleted cells did not reveal significant changes in lipid droplet binding or lipid synthetic pathway genes. The top downregulated gene in NDRG1 knockdown cells was Spot14 [42], which plays an incompletely understood regulatory role in stimulating the lipid synthetic process in breast cancer cells [43]. This result is consistent with a feedback inhibitory mechanism in cells that already have a high lipid level.

MCF7 cells normally express low levels of NDRG1 (Fig. 2b), and form few lipid droplets under normal culture conditions. NDRG1 expression is increased in MCF7 cells cultured under hypoxic conditions, which induces lipid droplet formation in other cell lines [44, 45]. While NDRG1 depletion has little effect on lipid droplet quantity in MCF7 cells under normal conditions, lipid droplet formation was stimulated by hypoxia (1% O_2,_ 24 h) and further enhanced 1.8-fold by NDRG1 depletion, both of which are conditions favoring lipogenesis (Fig. 5b). Two additional shRNAs confirmed that NDRG1 depletion causes lipid droplet formation in Hs578T cells, and electron microscopy affirmed the presence of lipid droplets at the ultrastructural level (Additional file 1: Figure S6C and Fig. 5d). Thus, multiple cell lines respond to NDRG1 depletion by increasing neutral lipid storage, especially under conditions that rely on a lipogenic metabolism.

In addition to silencing NDRG1, we generated stable SKBR3 cell lines overexpressing wild-type and truncation mutants lacking the N or C terminus (ΔN-NDRG1 and ΔC-NDRG1). The N and C termini of NDRG1 are predicted to be intrinsically disordered, and are known to be subject to post-translational modification. Notably, the N-terminus has been shown to be ubiquitinylated and SUMOylated, while the C-terminus is subject to extensive phosphorylation [36]. Guided by the structure of NDRG2 and its sequence alignment with NDRG1 [3], the N and C termini were excised to isolate their contribution to the lipid storage phenotype. Both ΔN-NDRG1 and ΔC-NDRG1 overexpressing SKBR3 cells exhibited increased lipid storage relative to the wild-type overexpressing control, but the increase was higher in ΔN-NDRG1 than in ΔC-NDRG1, showing 2.1-fold and 1.3-fold increases relative to the full-length control (Fig. 5c). These results suggest that the N-terminus of NDRG1 is particularly important in maintaining lipid homeostasis, and are consistent with the involvement of NDRG1 in minimizing lipid storage and promoting lipid homeostasis in breast cancer cells.

Dysregulated lipid metabolism has been linked with increased endoplasmic reticulum stress in breast cancer [46, 47] and increased lipid droplet formation is a conserved consequence of endoplasmic reticulum stress [47, 48]. Since we observed that NDRG1 regulates lipid droplet formation and since NDRG1 overexpression is induced in response to endoplasmic reticulum stress [49], we tested whether loss of NDRG1 impacted response to agents that induce endoplasmic reticulum stress. We found that cell death was enhanced twofold in NDRG1-depleted SKBR3 cells subjected to 48 h exposure with either thapsigargin or tunicamycin at 1 μM and 5 μg/ml, respectively (Additional file 1: Figure S7A). Importantly, treatment with the ATP competitive mTOR inhibitor Ku-0063794 (1 μM) had little effect on cell survival (Additional file 1: Figure S7A). In a series of doses spanning three orders of magnitude, NDRG1-depleted SKBR3 cells are more sensitive at each concentration, with reduced lethal dose 50% (LD50) values for each compound of ~ 100 fold for thapsigargin and ~ 75 fold for tunicamycin (Fig. 5e, f, Additional file 1: Figure S7). These results show that NDRG1 depletion sensitizes SKBR3 cells to agents with distinct mechanisms of endoplasmic reticulum stress induction [46] and suggest that NDRG1 functions in part to counteract endoplasmic reticulum stress.

### NDRG1 controls cellular fatty acid distribution

In order to better understand the impact of NDRG1 function on fatty acid trafficking and metabolism, BODIPY-labeled fatty acids were employed to trace their fate in living cells. Several BODIPY fatty acid conjugates are known to enter fatty acid metabolic pathways, including esterification to TAGs and phospholipids such as phosphatidylcholine, making them useful tools to study fatty acid uptake, metabolism, and distribution [32].

**Fig. 6:**
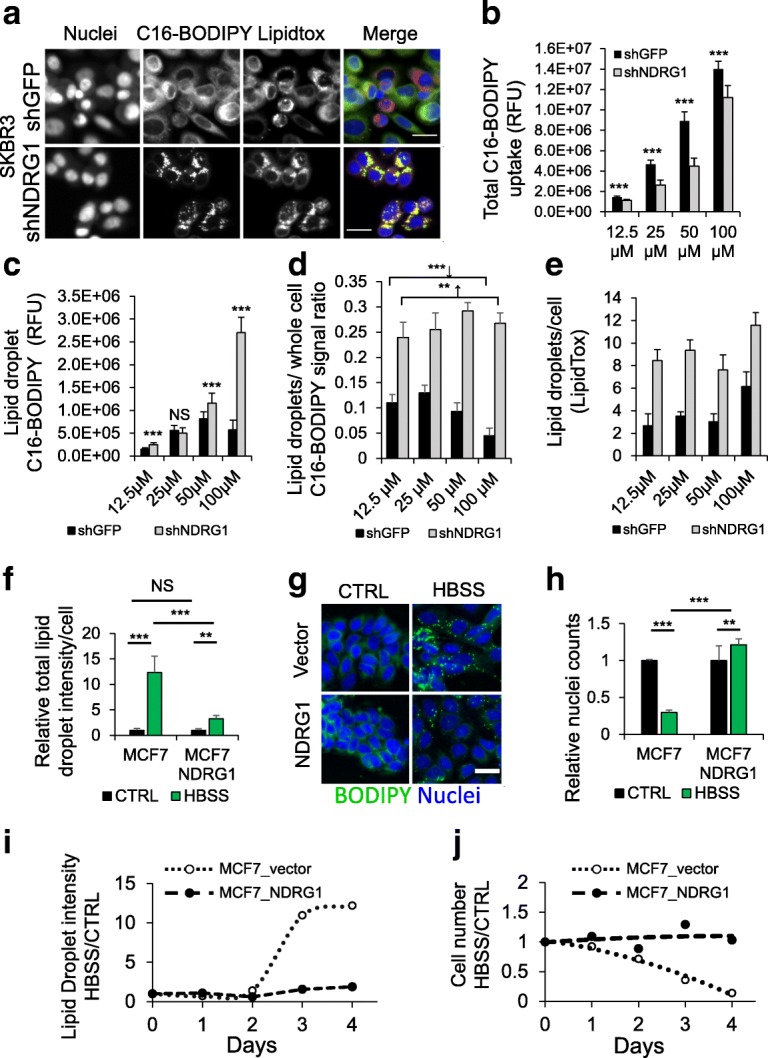
Fluorescent fatty acid tracers reveal NDRG1 suppresses fatty acid incorporation Into neutral lipids and storage in lipid droplets. **a** Fluorescence micrographs of stable NDRG1 SKBR3 +/− NDRG1 depletion fed the 16-carbon boron-dipyrromethene (BODIPY) palmitate (BODIPY C16–100 μM, 16 h), fixed. Lipid droplets and nuclei counterstained with LipidTox Deep Red and Hoechst, respectively. **b** Quantification of total BODIPY C16 uptake in SKBR3 cells +/− NDRG1 depletion. Graphs represent average total signal per cell at indicated tracer concentrations. **c** Quantification of lipid droplet specific BODIPY C16 by segmentation of small granules. **d** Relative distribution of BODIPY C16 signal representing droplet/whole cell signal. **e** LipidTox stained lipid droplet numbers represented as objects per cell. **f** Comparison of lipid droplet intensities of starved and fed controls at day 3 in stable BODIPY 558/568 C12 loaded MCF7 cells. Complete medium versus Hank’s buffered saline (HBSS). **g** BODIPY 493/503 counterstaining of NDRG1-FLAG overexpressing and control cells fed complete medium or HBSS for 3 days. **h** Analysis of cell survival due to starvation in stable BODIPY 558/568 C12-fed MCF7 cells transduced with NDRG1-FLAG or empty vector after 3 days of culture in HBSS. **i**, **j** Time course of changes in lipid droplet formation and cell count due to HBSS starvation in stable MCF7 cells transduced with the indicated constructs. Values represent HBSS/complete medium. All bar graphs represent mean +/− SD and comparisons were by two-sided *t* test. *N* = 12/group for all. Scale bars = 25 μm. CTRL, control

SKBR3 cells exhibited dramatic subcellular distributions of the fluorescent 16-carbon palmitate analog C16 BODIPY 493/503 that depended on NDRG1 expression and dosage of the tracer (Fig. 6a). Cells were fed a dilution series of albumin-coupled C16 BODIPY (12.5–100 μM), and total signal was quantified in segmented cells to compare overall cellular tracer uptake levels. Control and NDRG1-silenced SKBR3 cells showed dose-dependent increases in C16 BODIPY uptake (Fig. 6b), in line with experiments demonstrating that albumin-coupled oleic acid stimulates comparable increases in lipid droplet formation (Additional file 1: Figure S6A, B). NDRG1-depleted cells take up less of the tracer but overall signal intensity was similar at the highest dose (Fig. 6b), eliminating the possibility that NDRG1-silencing-dependent increases in neutral lipid storage are due to increased fatty acid uptake.

The intracellular distribution of C16 BODIPY showed surprising dose-dependent and NDRG1 status- dependent differences that suggest that NDRG1 helps distribute lipids within cells. C16 BODIPY localized to lipid droplets was quantified (Fig. 6c) and compared to overall uptake, yielding the fraction of tracer in droplets relative to that in the whole cell. In the NDRG1-depleted cell line, the relative balance of lipid droplet and total signal remained constant across all concentrations, and strong colocalization between lipid droplets and the tracer was maintained (Fig. 6a and d). Unexpectedly, as the dose of C16 BODIPY increased, the relative distribution of tracer signal changed in control cells, and a notable shift in tracer signal away from lipid droplets and into other parts of the cell was observed (Fig. 6a and d). In control cells, the proportion of tracer signal in lipid droplets actually decreased at the 50 μM and 100 μM doses, relative to the 25 μM dose, as total lipid droplet-specific signal gain was outpaced by total uptake and distribution to non-lipid droplet destinations (Fig. 6b–d). In contrast, the NDRG1-depleted cells consistently increased tracer signal in lipid droplets in accord with overall uptake (Fig. 6c). That the distribution of tracer was less dynamic in cells with decreased NDRG1 levels was confirmed by counter staining with LipidTOX Deep Red to quantify number and visualize localization of lipid droplets in C16-BODIPY-fed cells after 16 h. Although found in all treatment groups, LipidTOX-positive lipid droplets are largely distinct from C16 BODIPY stained droplets in control cells indicating redistribution of C16 BODIPY tracer after uptake. In NDRG1-depleted cells, a high degree of C16 BODIPY and LipidTOX Deep Red staining overlap was maintained at all C16 BODIPY levels (Fig. 6a). The average number of lipid droplets in each cell remained constant at 12.5, 25, and 50 μM C16 BODIPY doses, but increases in both cell lines at the 100 μM dose (shGFP 2-fold and shNDRG1 1.5-fold (Fig. 6e)). This shows that C16 BODIPY tracer feeding does not reduce lipid droplet numbers in either cell line, eliminating alternative explanations involving an NDRG1-dependent reduction in lipid droplet number at the 100 μM dose as grounds for the observed effect. Taken together, these results indicate that NDRG1 function influences pathways dictating the flow of fatty acids into storage and structural lipid pathways in breast cancer cells.

### Ectopic overexpression protects cells with low endogenous NDRG1 levels from starvation-mediated lipid droplet formation and cell death

NDRG1 expression is induced in response to metabolic limitations imposed by oxygen and iron deprivation, suggesting that it may play an important role in the altered metabolism associated with poorly perfused tumor microenvironments [17, 50–52]. Since lipid storage has been shown in a number of cell types to occur as a response to metabolic limitations of hypoxia [44] and glucose starvation [32, 53] and since our results showed that NDRG1 limits neutral lipid storage (Fig. 4), we tested whether NDRG1 expression protects breast cancer cells from conditions found in poorly perfused tumor microenvironments. MCF7 cells, which ordinarily express very low levels of NDRG1 (Fig. 3a) and do not produce high levels of lipids endogenously [21–23], were engineered to overexpress NDRG1 and empty vector control. These cells were pulsed overnight with the 12-carbon BODIPY fatty acid analog C12 BODIPY Red (100 μM), followed by a chase under tracer-free conditions. One population was cultured in HBSS for 3 days to mimic the nutrient starvation found in poorly perfused tumor microenvironments, and controls were cultured in complete medium. Both populations exhibited similar ubiquitous distributions of the tracer under normal culture conditions in complete medium. However, when NDRG1 overexpressing and vector control MCF7 cells were subjected to starvation in HBSS for 3 days, a striking shift in the tracer to lipid droplets was evident. The control cells, but not MCF7-NDRG1 cells, exhibited an acute shift in C12 BODIPY Red localization to lipid droplets. NDRG1 overexpressing cells appeared relatively unchanged over the course of starvation (Fig. 6f and g). In addition to attenuating lipid droplet formation, NDRG1 overexpression protected MCF7 cells from starvation-induced cell death. Starvation in HBSS for 72 h decreased control MCF7 cell numbers to 30% of fed control populations, while MCF7-NDRG1 cells maintained cell populations similar to fed controls under the same conditions (Fig. 6h). Similar results were observed with other cell types. SKBR3 cells expressing NDRG1 shRNAs displayed increased sensitivity to combined glucose, serum, and glutamine limitation (data not shown).

To understand the temporal nature of this process, and to rule out adverse effects due to the tracer, nutrient starvation was analyzed in unlabeled cells over a 4-day period. Interestingly, the spike in lipid droplet formation occurred between the 48 h and 72 h time points, and consistent with tracer results was coincident with the steepest period of decline in cell number in tracer-free conditions. Comparatively minor increases in lipid droplets were seen in MCF7-NDRG1 cells. Lipid droplet formation analysis showed 10-fold and 12-fold increases in lipid droplet staining intensity per cell in the control after 72 and 96 h of starvation, with comparatively small 1.5-fold and 1.8-fold increases in MCF7-NDRG1 cells at the same time points (Fig. 6i and j). Cell number decreases occurred in concert with lipid droplet formation in the control cells, and NDRG1 overexpressing cells remained at levels consistent with the fully fed control. Thus, NDRG1 overexpression both protects MCF7 cells from nutrient starvation induced cell death and attenuates starvation- induced lipid droplet formation. These results suggest that NDRG1 protects cancer cells from death in ill-perfused, nutrient-poor tumor microenvironments by optimizing lipid utilization. As NDRG1 is typically expressed in hypoxic microenvironments, however, it is likely that unlike most normal cells [32, 53], the lipids in breast cancer cells are ultimately likely to have fates other than beta oxidation.

## Discussion

Altered lipid metabolism is a common accessory pathway to the well-characterized increased dependence on glycolysis seen in breast cancer cells. This study defined a new function for the disease-associated protein NDRG1, and solidified its role as a negative prognostic marker in breast cancer. We showed that high *NDRG1* expression is a common feature of poor prognosis in breast cancer and that elevated expression is correlated with an aggressive metabolic gene signature and portends a high likelihood of disease recurrence and metastasis in several independent patient cohorts (Fig. 1a, b and Additional file 1: Figure S2). In vitro studies indicate that NDRG1 promotes optimal lipid composition and distribution in breast cancer cells, as depleting NDRG1 increases both lipid droplet and endosome formation and reduces viability, and overexpression protects cells from lipid droplet formation and starvation-induced cell death. Fluorescent fatty acid pulse-chase experiments show that NDRG1 plays a major role in directing the intracellular fate of fatty acids, favoring ubiquitous intracellular distribution in cells expressing high levels of NDRG1, or localization to lipid droplets and vesicles in cells with low levels of NDRG1. Although the precise role of NDRG1 may be impacted by other molecules or cells in the tumor microenvironment, these findings would appear to have significant implications for the understanding of lipid metabolism and cell survival under conditions including aerobic glycolysis (Warburg metabolism) and ill-perfused breast cancer microenvironments. The lipid management function described here is also consistent with the NDRG1 mutation-related defects in Schwann cell biology seen in CMT4D and impacts on cell size, which may be regulated directly by lipid availability [54].

Until now, elevated NDRG1 expression has alternately been referred to as both a biomarker of metastasis, and a metastasis suppressor, although in vivo experimental confirmation has been lacking. Our meta-analysis of the relationship between NDRG1 mRNA expression and prognosis in large populations of patients with breast cancer is definitive: overexpression of NDRG1 mRNA is a significant negative prognostic factor in breast cancer. Genomic expression analysis confirmed that elevated NDRG1 levels are associated with a metabolic gene expression profile that is a potent signature of aggressive disease in the most common solid tumor types, including breast cancer (Fig. 1e, f and data not shown). Similarly, the notion that the role of NDRG1 in the altered breast cancer metabolism associated with aggressive cells is in lipid management is supported by previous functional genomic studies [6, 8, 17] and that the lipid synthesis regulator Spot14 [42] is the most significantly downregulated gene in NDRG1 silenced SKBR3 cells. The fact that other genes related to lipid synthesis are not upregulated may be due to the high basal lipid synthesis activity of these cells. Expression of genes related to lipid-induced endoplasmic reticulum stress for example are already maximally induced in many human epidermal growth factor receptor 2 (HER2)-positive cell lines.

In addition to its binding partners, structural features suggest two means by which NDRG1 might participate in lipid trafficking in breast cancer cells. First, the inactive α/β hydrolase fold of NDRG1 could retain fatty acid binding activity in the substrate binding cleft or in a manner similar to the CGI-58 protein [55]. CGI-58 is an inactive α/β hydrolase recently shown to interact with lipid droplets through a lipid droplet anchor sequence composed of three clustered tryptophan residues. We note that among the NDRG family, only NDRG1 possesses a similar sequence of clustered tryptophans in alpha helix 6. Although the precise role of CGI-58 is incompletely understood, it appears to play some role in recruiting adipose triglyceride lipase (ATGL) to the surface of lipid droplets [55]. A similar mechanism for NDRG1 is consistent with our data, although more work would is needed to confirm this. Intriguingly, in a study examining NDRG1 and lipid binding, recombinant purified NDRG1 was shown to bind phosphatidylinositol 4-phosphate [6]. Second, NDRG1 encodes a consensus phosphopantetheine modification site. Direct evidence of this post-translational modification of NDRG1 has not been reported, but if modified, NDRG1 could support fatty acid trafficking or metabolism by reversibly binding fatty acids. One study examining the effect of NDRG1 on lipid trafficking in the epidermoid carcinoma cell line A431 showed NDRG1 silencing-altered multivesicular body morphology, reduced low density lipoprotein uptake due to mislocalization of the LDL receptor, and decreased cholesterol ester levels, while increasing ceramide levels [7]. Although the results differ, this study also points to altered lipid management in NDRG1-deficient cells. Further study is needed to determine whether NDRG1 directly binds additional fatty acids or complex lipids in a physiological setting.

NDRG1 has been implicated in divergent processes in a number of studies, complicating the interpretation of its function [56]. NDRG1 exhibits varied localization in tissues and cultured cell lines [57]. It is mainly cytoplasmic in breast cancer cells, but hypoxia or iron chelation causes dramatic redistribution of the pNDRG1 Ser330 pool to the nucleus in multiple cell types. Under both conditions, phospho-Thr346 NDRG1 remains cytoplasmic. These distinct modified forms may reconcile seemingly unrelated and even contradictory reports of NDRG1 function. In addition to lipid trafficking and metabolism, NDRG1 has also been implicated in DNA repair in the nucleus [8], and the phospho-Ser330 form may be the key player. In any event, fully deciphering NDRG1 regulation by post-translational modification will be extremely complex, as NDRG1 has been shown to be phosphorylated on at least 29 residues [36].

## Conclusions

The central role of NDRG1 in regulating fatty acid metabolic fate, neutral lipid storage, and cell viability in breast cancer cells establishes strong evidence of its function in cancer cell metabolism. In addition, these findings may be relevant to the NDRG1 mutation dependent Schwann cell pathology that leads to the characteristic demyelinating phenotype of CMT4D. Lipid synthesis is linked with glycolysis, and is a required component of transformed cell physiology [58]. Our previous work suggests that de novo fatty acid synthesis supports central carbon metabolism via redox coupling with malic enzyme 1 catalyzed conversion of malate to pyruvate in HER2-positive breast cancer cells [22]. In addition to the critical role of de novo fatty acid synthesis, Ras transformation, hypoxia, and TSC2 deficiency have all been linked to defects in fatty acid desaturation, necessitating alternative means of acquiring these fatty acids for the synthesis of structural lipids [59, 60]. Relatively little is known about factors regulating the fidelity of downstream lipid biosynthesis and trafficking pathways in cancer cells. NDRG1 expression correlates with glycolytic metabolism and poor outcomes in breast cancer. NDRG1 is expressed in all subtypes but is more likely to be constitutively expressed in ER-negative breast cancers, and dynamically expressed in response to microenvironment oxygen gradients in ER-positive breast cancers, suggesting it plays a smaller, but not insignificant, role in this setting. Our data place NDRG1 in the pathway dictating fatty acid utilization by the cell, downstream of both fatty acid synthesis and exogenous uptake, in which NDRG1 negatively regulates fatty acid storage in neutral lipids, and promotes alternative fates in cancer cells.

## Additional files

Additional file 1: Supplemental figures. (DOCX 2245 kb)

Additional file 2: Supplemental tables. (DOCX 30 kb)

Additional file 3: **Movie S1.** SKBR3 control. (AVI 3409 kb)

Additional file 4: **Movie S2.** SKBR3 control. (AVI 5866 kb)

Additional file 5: **Movie S3.** SKBR3 control. (AVI 4008 kb)

Additional file 6: **Movie S4.** SKBR3 NDRG1 knockdown. (AVI 2671 kb)

Additional file 7: **Movie S5.** SKBR3 NDRG1 knockdown. (AVI 3893 kb)

Additional file 8: **Movie S6.** SKBR3 NDRG1 knockdown. (AVI 2812 kb)

## Abbreviations

BODIPY: Boron-dipyrromethene
cDNA: Complementary DNA
CCLE: Cancer Cell Line Encyclopedia
CE: Cholesterol esters
CMT4D: Charcot-Marie-Tooth disease type 4D
Ctrl: Control
DFO: Deferoxamine
DMEM: Dulbecco’s modified Eagle’s medium
eGFP: Enhanced green fluorescent protein
ER/ESR1: Estrogen receptor
FBS: Fetal bovine serum
GAPDH: Glyceraldehyde-3-phosphate dehydrogenase
GFP: Green fluorescent protein
HBSS: Hank’s buffered saline solution
HER2: Human epidermal growth factor receptor 2
HMECs: Human mammary epithelial cells
LC-MS: Liquid chromatography-mass spectrometry
mRNA: Messenger RNA
NDRG1: N-myc downstream regulated gene
PBS: Phosphate-buffered saline
SDS-PAGE: Sodium dodecylsulfate polyacrylamide gel electrophoresis
shRNA: Short hairpin RNA
TAG: Triacylglycerols
TBS: Tris buffered saline

## References

[1] Kalaydjieva L, Gresham D, Gooding R, Heather L, Baas F, de Jonge R, Blechschmidt K, Angelicheva D, Chandler D, Worsley P (2000). N-myc downstream-regulated gene 1 is mutated in hereditary motor and sensory neuropathy-Lom. Am J Hum Genet.

[2] Hunter M, Angelicheva D, Tournev I, Ingley E, Chan DC, Watts GF, Kremensky I, Kalaydjieva L (2005). NDRG1 interacts with APO A-I and A-II and is a functional candidate for the HDL-C QTL on 8q24. Biochem Biophys Res Commun.

[3] Hwang J, Kim Y, Kang HB, Jaroszewski L, Deacon AM, Lee H, Choi W-C, Kim K-J, Kim C-H, Kang BS (2011). Crystal structure of the human N-Myc downstream-regulated gene 2 protein provides insight into its role as a tumor suppressor. J Biol Chem.

[4] Shi X-H, Larkin JC, Chen B, Sadovsky Y. The expression and localization of N-myc downstream-regulated gene 1 in human trophoblasts. PLoS One. 2013;8 10.1371/journal.pone.0075473 PMC3774633 24066183

[5] Croessmann S, Wong HY, Zabransky DJ, Chu D, Mendonca J, Sharma A, Mohseni M, Rosen DM, Scharpf RB, Cidado J (2015). NDRG1 links p53 with proliferation-mediated centrosome homeostasis and genome stability. Proc Natl Acad Sci U S A.

[6] Kachhap SK, Faith D, Qian DZ, Shabbeer S, Galloway NL, Pili R, Denmeade SR, DeMarzo AM, Carducci MA (2007). The N-myc down regulated gene1 (NDRG1) is a Rab4a effector involved in vesicular recycling of E-cadherin. PLoS One.

[7] Pietiäinen V, Vassilev B, Blom T, Wang W, Nelson J, Bittman R, Bäck N, Zelcer N, Ikonen E (2013). NDRG1 functions in LDL receptor trafficking by regulating endosomal recycling and degradation. J Cell Sci.

[8] Weiler M, Blaes J, Pusch S, Sahm F, Czabanka M, Luger S, Bunse L, Solecki G, Eichwald V, Jugold M (2014). mTOR target NDRG1 confers MGMT-dependent resistance to alkylating chemotherapy. Proc Natl Acad Sci U S A.

[9] Bandyopadhyay S, Pai SK, Gross SC, Hirota S, Hosobe S, Miura K, Saito K, Commes T, Hayashi S, Watabe M (2003). The Drg-1 gene suppresses tumor metastasis in prostate cancer. Cancer Res.

[10] Bandyopadhyay S, Pai SK, Hirota S, Hosobe S, Takano Y, Saito K, Piquemal D, Commes T, Watabe M, Gross SC (2004). Role of the putative tumor metastasis suppressor gene Drg-1 in breast cancer progression. Oncogene.

[11] Guan RJ, Ford HL, Fu Y, Li Y, Shaw LM, Pardee AB (2000). Drg-1 as a differentiation-related, putative metastatic suppressor gene in human colon cancer. Cancer Res.

[12] Hu Z, Fan C, Livasy C, He X, Oh DS, Ewend MG, Carey LA, Subramanian S, West R, Ikpatt F (2009). A compact VEGF signature associated with distant metastases and poor outcomes. BMC Med.

[13] Ring BZ, Seitz RS, Beck R, Shasteen WJ, Tarr SM, Cheang MCU, Yoder BJ, Budd GT, Nielsen TO, Hicks DG (2006). Novel prognostic immunohistochemical biomarker panel for estrogen receptor-positive breast cancer. J Clin Oncol Off J Am Soc Clin Oncol.

[14] Chen X, Iliopoulos D, Zhang Q, Tang Q, Greenblatt MB, Hatziapostolou M, Lim E, Tam WL, Ni M, Chen Y (2014). XBP1 promotes triple-negative breast cancer by controlling the HIF1α pathway. Nature.

[15] Piperi C, Adamopoulos C, Papavassiliou AG (2016). XBP1: a pivotal transcriptional regulator of glucose and lipid metabolism. Trends Endocrinol Metab.

[16] Sood A, Miller AM, Brogi E, Sui Y, Armenia J, McDonough E, Santamaria-Pang A, Carlin S, Stamper A, Campos C, et al. Multiplexed immunofluorescence delineates proteomic cancer cell states associated with metabolism. JCI Insight. 2016;1 10.1172/jci.insight.87030 PMC4863708 27182557

[17] Askautrud HA, Gjernes E, Gunnes G, Sletten M, Ross DT, Børresen-Dale AL, Iversen N, Tranulis MA, Frengen E (2014). Global gene expression analysis reveals a link between NDRG1 and vesicle transport. PLoS One.

[18] Cancer Genome Atlas Network. Comprehensive molecular portraits of human breast tumours. Nature. 2012;490(7418):61–70. 10.1038/nature11412 PMC3465532 23000897

[19] Ahn SG, Park JT, Lee HM, Lee HW, Jeon TJ, Han K, Lee SA, Dong SM, Ryu YH, Son EJ (2014). Standardized uptake value of ^18^F-fluorodeoxyglucose positron emission tomography for prediction of tumor recurrence in breast cancer beyond tumor burden. Breast Cancer Res.

[20] Liberti MV, Locasale JW (2016). The Warburg effect: how does it benefit cancer cells?. Trends Biochem Sci.

[21] Baumann J, Sevinsky C, Conklin DS. Lipid biology of breast cancer. Biochim Biophys Acta. 2013;1831(10):1509–17. 10.1016/j.bbalip.2013.03.011 PMC3926128 23562840

[22] Kourtidis A, Jain R, Carkner RD, Eifert C, Brosnan MJ, Conklin DS (2010). An RNA interference screen identifies metabolic regulators NR1D1 and PBP as novel survival factors for breast cancer cells with the ERBB2 signature. Cancer Res.

[23] Menendez JA, Lupu R (2007). Fatty acid synthase and the lipogenic phenotype in cancer pathogenesis. Nat Rev Cancer.

[24] Hitoshi Y, Lorens J, Kitada SI, Fisher J, LaBarge M, Ring HZ, Francke U, Reed JC, Kinoshita S, Nolan GP (1998). Toso, a cell surface, specific regulator of Fas-induced apoptosis in T cells. Immunity.

[25] Hannon GJ, Sun P, Carnero A, Xie LY, Maestro R, Conklin DS, Beach D (1999). MaRX: an approach to genetics in mammalian cells. Science.

[26] Ejsing CS, Duchoslav E, Sampaio J, Simons K, Bonner R, Thiele C, Ekroos K, Shevchenko A (2006). Automated identification and quantification of glycerophospholipid molecular species by multiple precursor ion scanning. Anal Chem.

[27] Ekroos K, Chernushevich IV, Simons K, Shevchenko A (2002). Quantitative profiling of phospholipids by multiple precursor ion scanning on a hybrid quadrupole time-of-flight mass spectrometer. Anal Chem.

[28] Shaner RL, Allegood JC, Park H, Wang E, Kelly S, Haynes CA, Sullards MC, Merrill AH (2009). Quantitative analysis of sphingolipids for lipidomics using triple quadrupole and quadrupole linear ion trap mass spectrometers. J Lipid Res.

[29] Liebisch G, Binder M, Schifferer R, Langmann T, Schulz B, Schmitz G (2006). High throughput quantification of cholesterol and cholesteryl ester by electrospray ionization tandem mass spectrometry (ESI-MS/MS). Biochim Biophys Acta.

[30] Cerami E, Gao J, Dogrusoz U, Gross BE, Sumer SO, Aksoy BA, Jacobsen A, Byrne CJ, Heuer ML, Larsson E (2012). The cBio Cancer Genomics Portal: an open platform for exploring multidimensional cancer genomics data. Cancer Discov.

[31] Györffy B, Lanczky A, Eklund AC, Denkert C, Budczies J, Li Q, Szallasi Z (2010). An online survival analysis tool to rapidly assess the effect of 22,277 genes on breast cancer prognosis using microarray data of 1,809 patients. Breast Cancer Res Treat.

[32] Rambold AS, Cohen S, Lippincott-Schwartz J (2015). Fatty acid trafficking in starved cells: regulation by lipid droplet lipolysis, autophagy and mitochondrial fusion dynamics. Dev Cell.

[33] Jin R, Liu W, Menezes S, Yue F, Zheng M, Kovacevic Z, Richardson DR (2014). The metastasis suppressor NDRG1 modulates the phosphorylation and nuclear translocation of β-catenin through mechanisms involving FRAT1 and PAK4. J Cell Sci.

[34] van 't Veer LJ, Dai H, van de Vijver MJ, He YD, Hart AAM, Mao M, Peterse HL, van der Kooy K, Marton MJ, Witteveen AT (2002). Gene expression profiling predicts clinical outcome of breast cancer. Nature.

[35] Barretina J, Caponigro G, Stransky N, Venkatesan K, Margolin AA, Kim S, Wilson CJ, Lehár J, Kryukov GV, Sonkin D (2012). The Cancer Cell Line Encyclopedia enables predictive modelling of anticancer drug sensitivity. Nature.

[36] Hornbeck PV, Zhang B, Murray B, Kornhauser JM, Latham V, Skrzypek E (2015). PhosphoSitePlus, 2014: mutations, PTMs and recalibrations. Nucleic Acids Res.

[37] Murray JT, Campbell DG, Morrice N, Auld GC, Shpiro N, Marquez R, Peggie M, Bain J, Bloomberg GB, Grahammer F (2004). Exploitation of KESTREL to identify NDRG family members as physiological substrates for SGK1 and GSK3. Biochem J.

[38] Sahni S, Bae D-H, Lane DJR, Kovacevic Z, Kalinowski DS, Jansson PJ, Richardson DR (2014). The metastasis suppressor, N-myc downstream-regulated gene 1 (NDRG1), inhibits stress-induced autophagy in cancer cells. J Biol Chem.

[39] Fang BA, Kovačević Ž, Park KC, Kalinowski DS, Jansson PJ, Lane DJR, Sahni S, Richardson DR (2014). Molecular functions of the iron-regulated metastasis suppressor, NDRG1, and its potential as a molecular target for cancer therapy. Biochim Biophys Acta.

[40] Walther TC, Farese RV (2012). Lipid droplets and cellular lipid metabolism. Annu Rev Biochem.

[41] Baumann JM, Kokabee L, Wang X, Sun Y, Wong J, Conklin DS. Metabolic assays for detection of neutral fat stores. Bio Protoc. 2015;5(12) 10.21769/bioprotoc.1511 PMC4957654 27453913

[42] Kinlaw WB, Quinn JL, Wells WA, Roser-Jones C, Moncur JT (2006). Spot 14: a marker of aggressive breast cancer and a potential therapeutic target. Endocrinology.

[43] Park S, Hwang I-W, Makishima Y, Perales-Clemente E, Kato T, Niederländer NJ, Park EY, Terzic A (2013). Spot14/Mig12 heterocomplex sequesters polymerization and restrains catalytic function of human acetyl-CoA carboxylase 2. J Mol Recognit.

[44] Bensaad K, Favaro E, Lewis CA, Peck B, Lord S, Collins JM, Pinnick KE, Wigfield S, Buffa FM, Li J-L (2014). Fatty acid uptake and lipid storage induced by HIF-1α contribute to cell growth and survival after hypoxia-reoxygenation. Cell Rep.

[45] Boström P, Magnusson B, Svensson P-A, Wiklund O, Borén J, Carlsson LMS, Ståhlman M, Olofsson S-O, Hultén LM (2006). Hypoxia converts human macrophages into triglyceride-loaded foam cells. Arterioscler Thromb Vasc Biol.

[46] Baumann J, Wong J, Sun Y, Conklin DS, Palmitate-induced ER (2016). stress increases trastuzumab sensitivity in HER2/neu-positive breast cancer cells. BMC Cancer.

[47] Volmer R, Ron D (2015). Lipid-dependent regulation of the unfolded protein response. Curr Opin Cell Biol.

[48] Fei W, Wang H, Fu X, Bielby C, Yang H (2009). Conditions of endoplasmic reticulum stress stimulate lipid droplet formation in Saccharomyces cerevisiae. Biochem J.

[49] Kokame K, Kato H, Miyata T (1996). Homocysteine-respondent genes in vascular endothelial cells identified by differential display analysis GRP78/BiP and novel genes. J Biol Chem.

[50] Cangul H (2004). Hypoxia upregulates the expression of the NDRG1 gene leading to its overexpression in various human cancers. BMC Genet.

[51] Lai L-C, Su Y-Y, Chen K-C, Tsai M-H, Sher Y-P, Lu T-P, Lee C-Y, Chuang EY (2011). Down-regulation of NDRG1 promotes migration of cancer cells during reoxygenation. PLoS One.

[52] Lane DJR, Saletta F, Rahmanto YS, Kovacevic Z, Richardson DR (2013). N-myc downstream regulated 1 (NDRG1) is regulated by eukaryotic initiation factor 3a (eIF3a) during cellular stress caused by iron depletion. PLoS One.

[53] Singh R, Kaushik S, Wang Y, Xiang Y, Novak I, Komatsu M, Tanaka K, Cuervo AM, Czaja MJ (2009). Autophagy regulates lipid metabolism. Nature.

[54] Rao MJ, Srinivasan M, Rajasekharan R. Cell size is regulated by phospholipids and not by storage lipids in Saccharomyces cerevisiae. Curr Genet. 2018;1831(10):1509–17. 10.1007/s00294-018-0821-0 10.1007/s00294-018-0821-0 29536156

[55] Boeszoermenyi A, Nagy HM, Arthanari H, Pillip CJ, Lindermuth H, Luna RE, Wagner G, Zechner R, Zangger K, Oberer M (2015). Structure of a CGI-58 motif provides the molecular basis of lipid droplet anchoring. J Biol Chem.

[56] Ellen TP, Ke Q, Zhang P, Costa M (2008). NDRG1, a growth and cancer related gene: regulation of gene expression and function in normal and disease states. Carcinogenesis.

[57] Lachat P, Shaw P, Gebhard S, van Belzen N, Chaubert P, Bosman FT (2002). Expression of NDRG1, a differentiation-related gene, in human tissues. Histochem Cell Biol.

[58] Pavlova NN, Thompson CB (2016). The emerging hallmarks of cancer metabolism. Cell Metab.

[59] Kamphorst JJ, Cross JR, Fan J, de Stanchina E, Mathew R, White EP, Thompson CB, Rabinowitz JD (2013). Hypoxic and Ras-transformed cells support growth by scavenging unsaturated fatty acids from lysophospholipids. Proc Natl Acad Sci U S A.

[60] Young RM, Ackerman D, Quinn ZL, Mancuso A, Gruber M, Liu L, Giannoukos DN, Bobrovnikova-Marjon E, Diehl JA, Keith B (2013). Dysregulated mTORC1 renders cells critically dependent on desaturated lipids for survival under tumor-like stress. Genes Dev.

