# Supplementary material for: NDRG1 regulates neutral lipid metabolism in breast cancer cells
Source: Breast Cancer Res. 2018 Jun 14;20:55. doi: 10.1186/s13058-018-0980-4 (PMC6001025; doi:10.1186/s13058-018-0980-4)
Supplement: Supplementary file 1 — Supplemental figures. (DOCX 2245 kb) [file 13058_2018_980_MOESM1_ESM.docx]

Additional file 1

**TCGA Breast Cancer mRNA expression analysis:**

The breast cancer specific mRNA expression characteristics were examined for genes coexpresssed or anti-correlated with NDRG1 expression in order to better understand relationships between NDRG1 and markers reflecting intrinsic molecular subtypes. In one analysis, cases were divided into altered and unaltered based on significant alterations in genomic or protein phosphorylation characteristics to find genes with the most commonly positively or negatively correlated mRNA expression in breast cancer (described below). Candidate genes were ranked by Q-scores and selected genes with established relationships with luminal or non-luminal breast cancer were examined. Luminal and basal breast cancer subtype associated genes were assessed, and values are expressed as fold changes compared to the unaltered group.

Pairwise correlation between NDRG1 and all other genes in the TCGA breast cancer data asset was also downloaded and analyzed (Supplemental Dataset 1).

**TCGA 18 cancer type meta-analysis:**

The cBio portal was accessed in order to analyze global gene expression patterns across 18 human solid tumor types (Cerami et al., 2012). Cancers included Bladder cancer, Cervical cancer, Stomach cancer, Head/neck cancer, Glioblastoma, Pheochromocytoma and Paraganglioma, Lung adenocarcinoma, Lung squamous cell carcinoma, High grade Serous Ovarian cancer, Pancreatic ductal adenocarcinoma, Prostate adenocarcinoma, Sarcoma, Uterine cancer, Renal papillary cell carcinoma, Breast cancer, Renal clear cell carcinoma, Esophageal cancer, and cutaneous Melanoma. To identify those genes whose expression reflects the physiology associated with increased NDRG1 levels, two selection criteria were established. First, in each cancer type, genes with mRNA Pearson’s product moment correlation coefficient (R) ≥ 0.40 were selected to retrieve a list of NDRG1 associated genes. Second, while correlations within each individual cancer may yield important insights, we reasoned that a correlated gene signature shared between many cancers would eliminate intra-disease correlations reflecting cell of origin (e.g. Keratin genes), but unrelated to NDRG1 function. Therefore, genes meeting the minimum correlation value in at least 33% of cancers analyzed are considered pancancer *NDRG1* correlated genes. This approach should reveal common elements of an underlying biological process, illuminating the physiological role of a poorly characterized gene (Complete data are found in Supplemental Dataset 1).

**TCGA NDRG1 T346 expression level analysis:**

In addition to mRNA levels, the TCGA analyzed NDRG1 phosphorylation on the decapeptide triple repeat encoded threonine 346. The approach used to analyze mRNA levels of genes in altered and unaltered cases was used to assess coexpression of proteins analyzed by reverse phase protein array.

**Oncoprint statistics**. Alteration defined as mRNA or RPPA z-score > 1.5, amplification, deletion, or missense mutation.

**Survival analysis.**

To assess the relationship between NDRG1 mRNA expression and breast cancer patient disease and metastasis free survival, we assessed a number of publicly available gene expression data sets. One analysis queried 23 data sets representing 3355 unique breast cancer patients. The online tool KM plotter was used to establish query criteria and generate KM plots, hazard ratios, 95% confidence intervals, and p-values (Györffy et al., 2010). Because NDRG1 alterations occur in ~25% of breast cancer patients analyzed in by the TCGA, survival curves were split based on the upper quartile of NDRG1 expression. Each individual accession was analyzed by Kaplan Meier survival curves and Log-rank tests in five year relapse-free survival analysis independently or as an aggregate cohort. The often coamplified oncogene *MYC* and common marker of hypoxia *SLC2A1* (GLUT1) were also analyzed in the aggregate cohort.

Additional cohorts were analyzed by accessing independent patient cohorts with the online tool SurExpress (<http://bioinformatica.mty.itesm.mx:8080/Biomatec/SurvivaX.jsp>). NDRG1, NDRG1 + MYC, or the forty-two member NDRG1 associated gene signature were queried for relationships with adverse outcomes (metastasis-free survival, or recurrence free survival). Again, because NDRG1 is altered in roughly 25% of all TCGA cases, the patients were split by quartiles for Kaplan-Meier analysis and Logrank test to understand the relationship between progressive increases in expression of the explanatory variables and the clinical outcome in question. P values of the logrank test and hazard ratios were used to assess the strength of the association between any given gene or genes and the clinical outcome. The genes were also analyzed as continuous variables by Cox proportional hazards modeling to understand the relative importance of each explanatory variable to the survival model (e.g, comparison of NDRG1 and MYC). The TCGA, Van’t Veer, and Metabase breast cancer cohorts were analyzed. *MYC* and *SLC2A1* (GLUT1) were also analyzed in the Van’t Veer cohort.

**Cancer Cell Line Encyclopedia breast cancer cell line mRNA expression profiles.**

The mRNA expression of *NDRG1* in > 1000 cell lines was downloaded from the Broad Institute CCLE portal: <https://portals.broadinstitute.org/ccle/home>. Breast cancer cell lines were filtered, ranked according to expression level, and plotted to evaluate the range of expression in characterized cell lines. Cell lines chosen for *in vitro* studies are indicated.

**Estrogen receptor status correlation.**

*NDRG1* and *ESR1* expression from the Van’t Veer cohort was downloaded and dichotomized based on ER status. ER stats was not available from the Metabase cohort, therefore continuous measures were obtained and analyzed for correlation. The levels of NDRG1 expression were compared by two-sided Student’s T-test. ESR1 and NDRG1 were also analyzed for correlation by Pearson product-moment correlation coefficient in both the meta-base (n=1901) and Van’t Veer cohorts (n=295) and t values were analyzed by two-sided T-test to determine statistical significance. All *ESR1/NDRG1* correlation analysis was performed using SigmaPlot software.


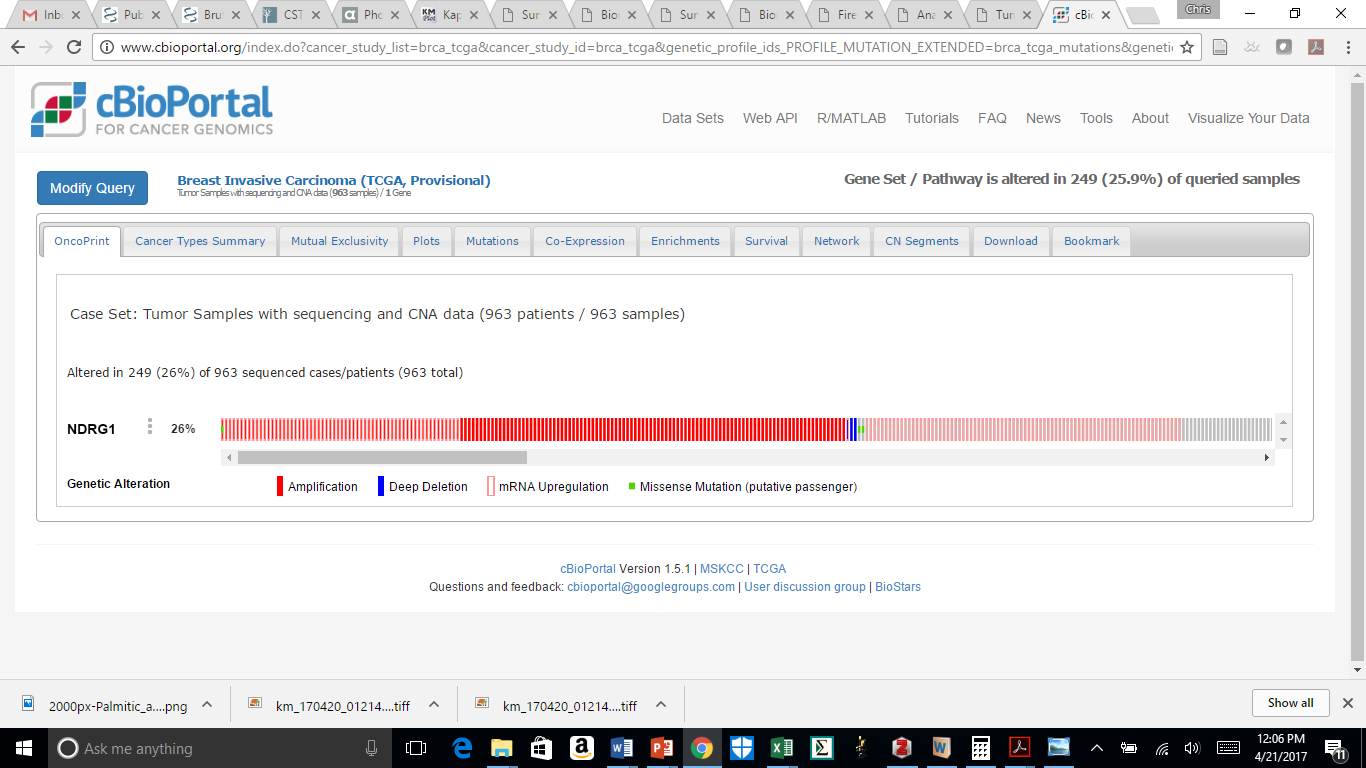

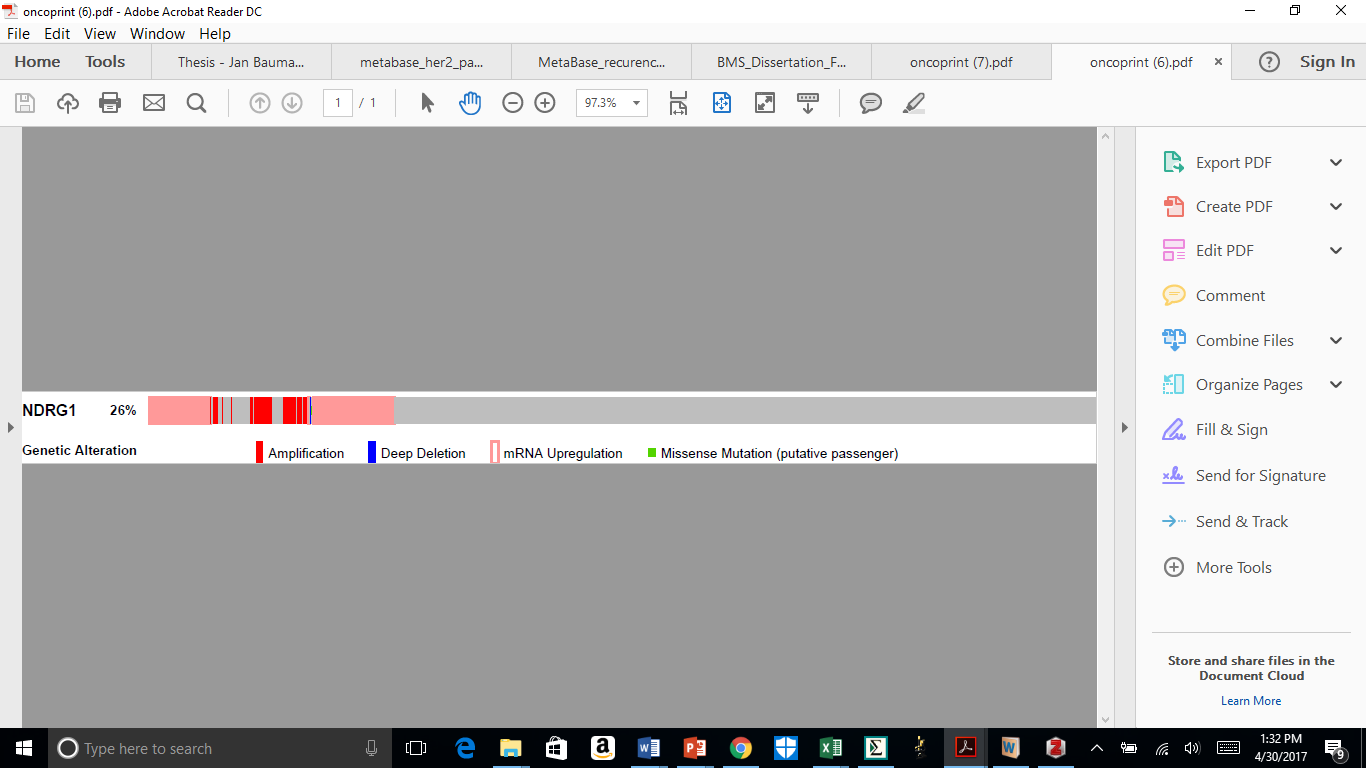


NDRG1 Genomic Alteration Frequency: 26%

mRNA overexpression

Gene amplification

Gene deletion – homozygous loss

Missense mutation

Legend

NDRG1

**Figure S1. NDRG1 genomic alterations in the TCGA breast cancer data set** - copy number, expression (+/-1.5 fold) and missense mutations.


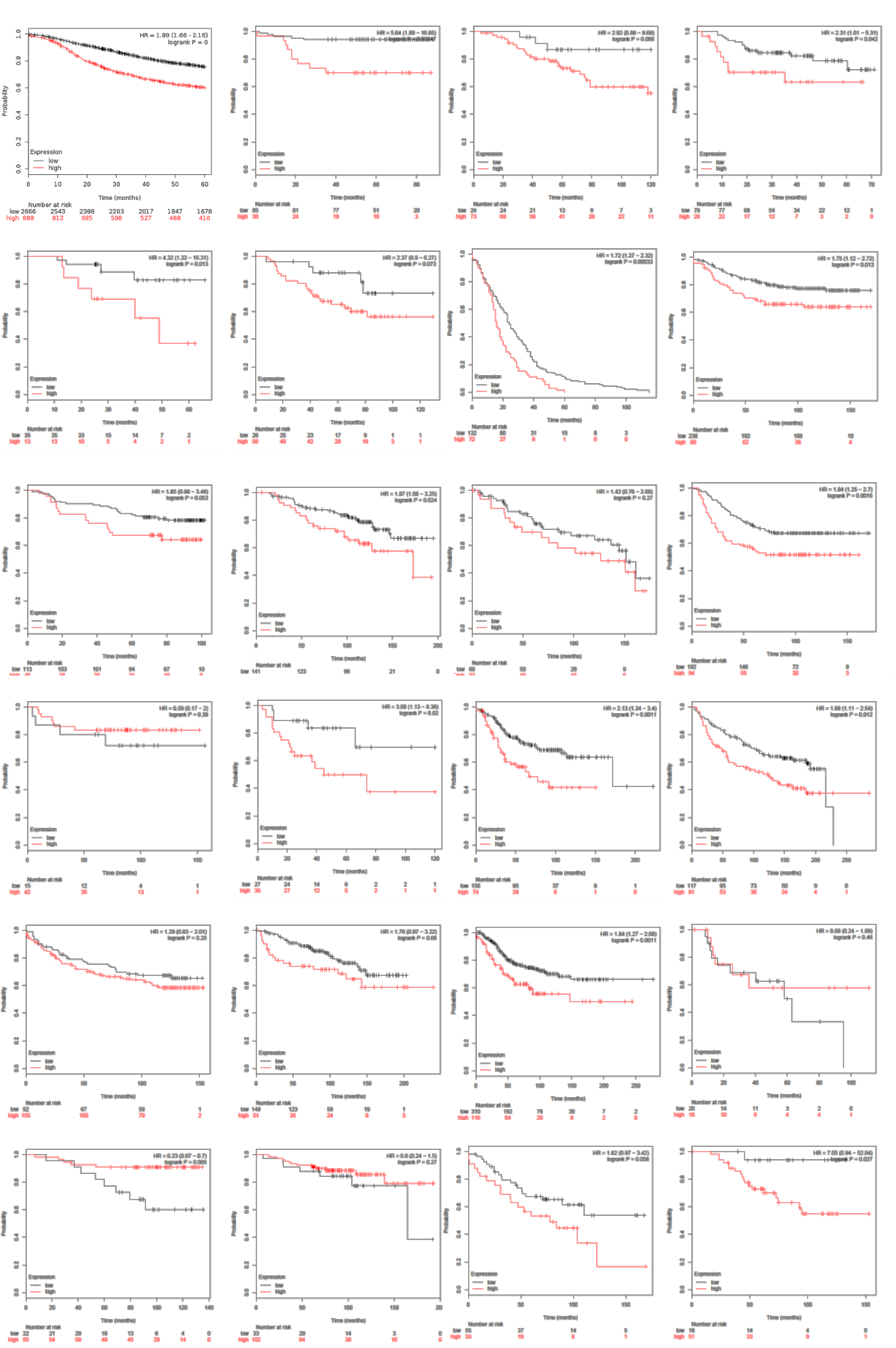


**Figure S2: Individual cohorts show *NDRG1* Expression is commonly associated with adverse breast cancer outcomes.** Kaplan Meier analysis of the meta-analysis cohort was divided into 23 distinct breast cancer cohorts to reflect the trends associated with each study. 19/23 cohorts exhibit significant or near significant poor prognosis trends, 3/23 show no signs of separation, and one cohort exhibits favorable prognosis in the NDRG1 high group. Patients are split by Upper quartile and lower three quartiles as in Figure 3.2


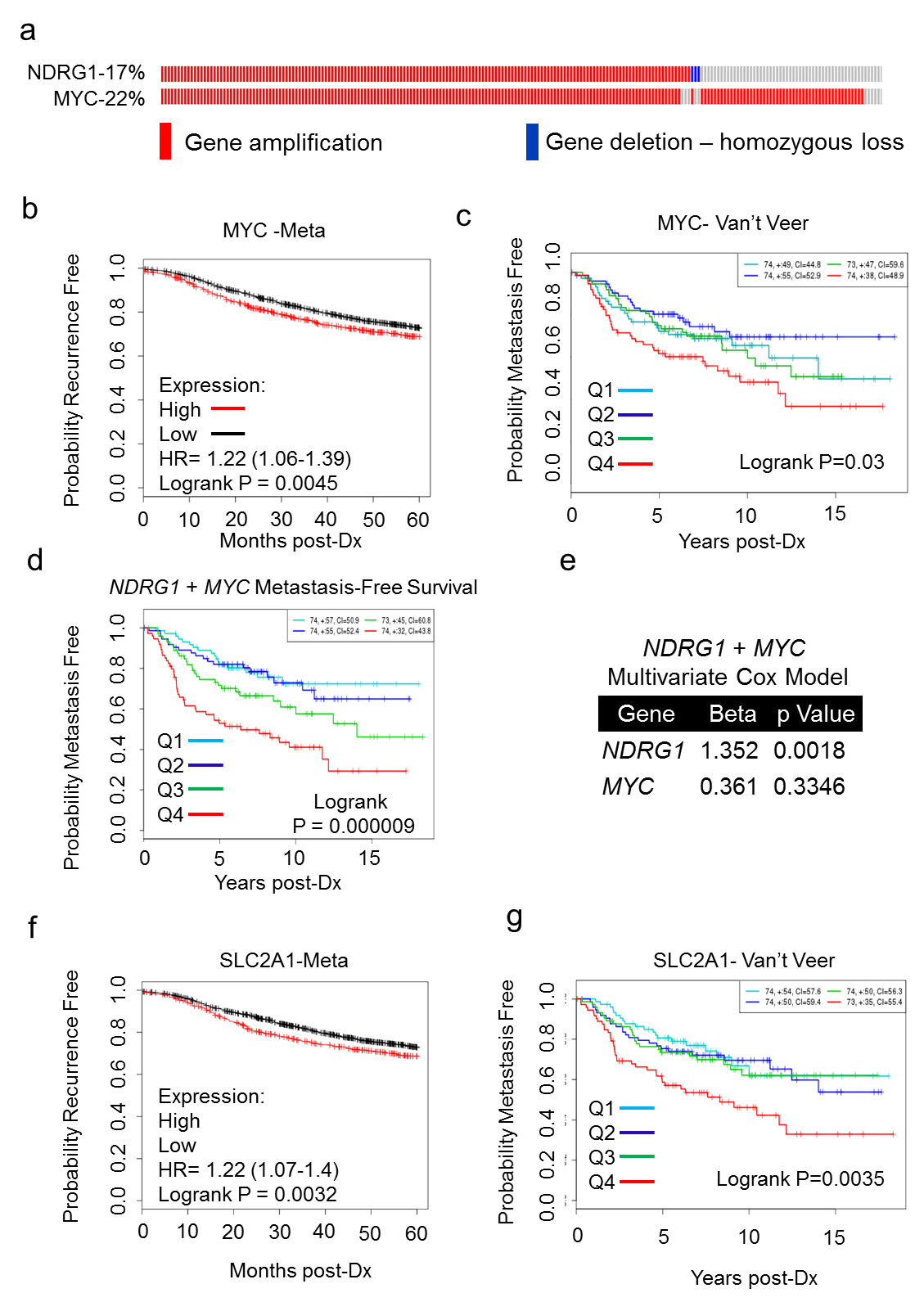
‘

**Figure S3: Analysis of *MYC* and *SLC2A1* expression and survival characteristics.** (A) Co amplification of *NDRG1* and *MYC* in the TCGA breast cancer cohort (n=962). (B, C) Kaplan Meier analysis of *MYC* in the meta-analysis and Van’t Veer cohorts. (D) Kaplan Meier curves of multivariate *NDRG1* and *MYC* analysis in the Van’t Veer cohort. (E) Cox proportional hazards risk model parameters and p-values from combined NDRG1 and MYC analysis. (F, G) Kaplan Meier analysis of *SLC2A1* in the meta-analysis and Van’t Veer cohorts. Meta-anlaysis, n=3554 and Van’t Veer n= 295.

**Hypoxia, DFO treatment and NDRG1 expression and localization studies:**

Increased total NDRG1 protein expression due to 24 hrs Deferoxamine or hypoxia treatment was assessed by immunofluorescence analysis. Deferoxamine and hypoxia were found to selectively induce translocation of most of the pNDRG1 S330 pool to the nucleus, whereas the pNDRG1 T346 pool remained largely unchanged. In order to quantify translocation, ImageJ was used to generate masks of the nuclear and non-nuclear compartments of each cell. This was conducted on images collected by widefield microscopy at 20X magnification. First, a whole cell mask was generated using phalloidin counterstain. These masks were augmented by the hole filling function in the binary command module in order to eliminate intracellular voids created in phalloidin low areas. Next, Hoechst counter stain was used to generate a nuclear mask. To generate the non-nuclear area mask, the nuclear mask was dilated by 5 pixels and subtracted from the whole cell mask. This effectively removed nuclear area and a very thin halo surrounding the nucleus in order to minimize signal at the interface of the segmented regions. The original nuclear mask was eroded by 5 pixels to achieve the same effect. Each mask was applied to stained images using the region of interest manager, and standard image metrics including average expression in the segmented regions were obtained. The average signal or integrated densities in each compartment was computed and expressed as a ratio of nuclear:non-nuclear signal. Ratios of greater than one indicate a predominantly nuclear localization, whereas ratios less than one are considered predominantly non-nuclear. Integrated density was analyzed to represent the quantity of NDRG1 species per compartment.

The sensitivity of the pNDRG1 T346 puncta in SKBR3 cells was analyzed in response to various drugs. Cells were treated with thapsigargin (1 μM) or tunicamycin (5 mg/mL) for 15 minutes and compared to vehicle control (DMSO only).


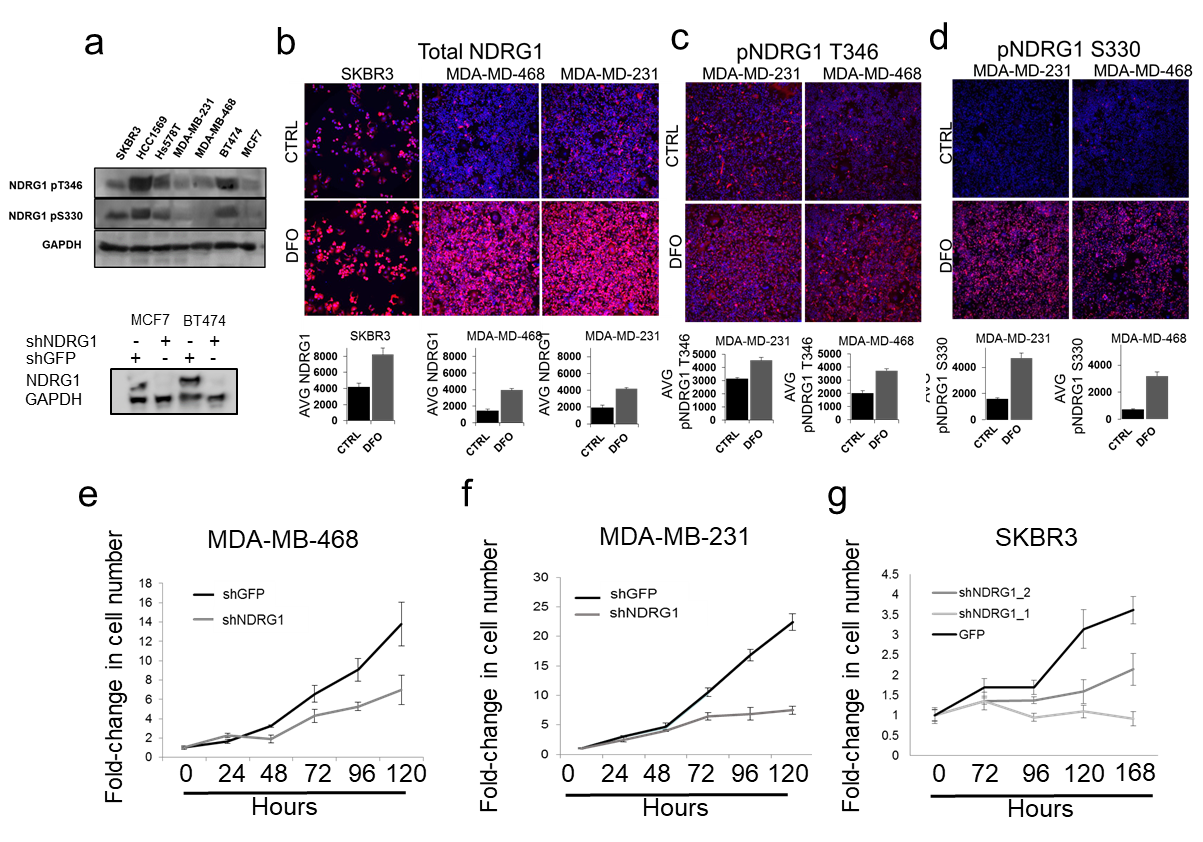

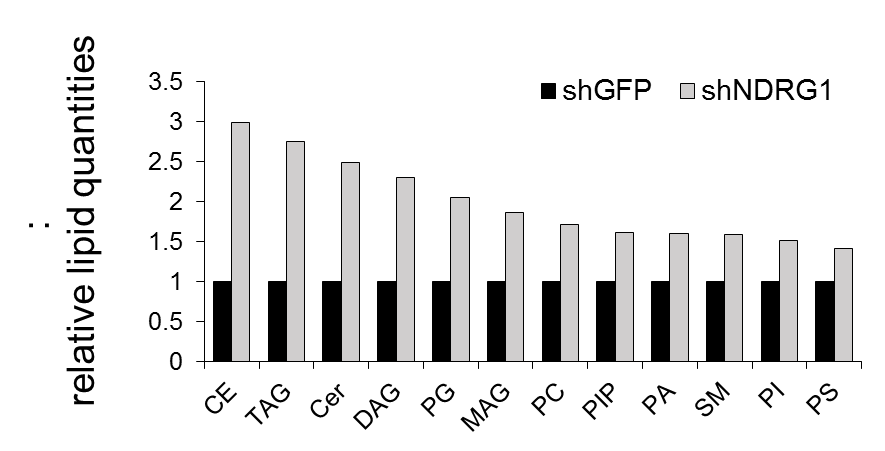


**Figure S4. NDRG1 phosphorylation levels in breast cancer cell lines. DFO treatment increases total and phosphorylated NDRG1 protein levels. NDRG1 depletion reduces growth rates in cultured cells.** Related to Figures 2B,D,E and 3A,B. (A) Immunoblots of phosphorylated forms of NDRG1 in seven cell lines. Knock down efficiency in MCF7 and BT474. (B) SKBR3, MDA-MB-468, and MDA-MB-231 cells were treated with 100 μM Deferoxamine (DFO) overnight (16 hrs), fixed, permeablized, probed with αNDRG1 antibody counterstained with Hoechst. N= 6/group. (C,D) Cells were grown and treated as in (E), and the indicated phosphorylated NDRG1 residues were probed. N= 6/group. (E-G) Growth curves of stable cell lines expressing indicated short hairpin RNAs targeting NDRG1 or GFP were plated at low density, fixed, Hoechst stained, and counted at the indicated times. All population sizes are represented as fold increases relative to time zero, n=3/group. All P<0.00001, 2-sided Student’s T-test. All graphs represent mean intensity +/- standard deviation.

**Figure S5. Shotgun Lipidomics Analysis** – related to Figure 4A. (A) Aggregate lipid species were quantified in an independent run of shotgun LC MS/MS lipidomics. Bar graphs represent average fold changes in NDRG1 depleted cells normalized to shGFP expressing cells. N=3.

**Proliferation assays and lipid droplet counts by automated image analysis.**

For cell proliferation studies, drug selected cells were plated at low densities in 96 well microtiter plates. Viable cell counts were determined by Trypan blue dye exclusion assay (Countess – Thermo Fisher Scientific). Cells with rapid cell division rates (~ 24 hrs: MDA-MB231, MDA-MB-468) were plated at 2,000 viable cells per well, all other cell lines were plated at 5,000 viable cells per well.

Cells were imaged in the IN Cell Analyzed 2200 (GE Healthcare). A minimum of four images per well from a minimum of 3 wells were collected for each condition, and data were summarized at the well level for statistical comparisons. Exposure times were set to allow quantitative analysis of intensity using statistical tests comparing brightness of objects (e.g., lipid droplets). Images were analyzed using GE Healthcare In Cell Analyzer software granule and nuclei counting algorithms. For lipid droplet granules, sensitivity was set to the most stringent threshold to reduce nonspecific granule counting. Particle size was set to the range 0.1-2 µm^2^. Nuclei were also counted - minimum size 40 µm^2^.

**
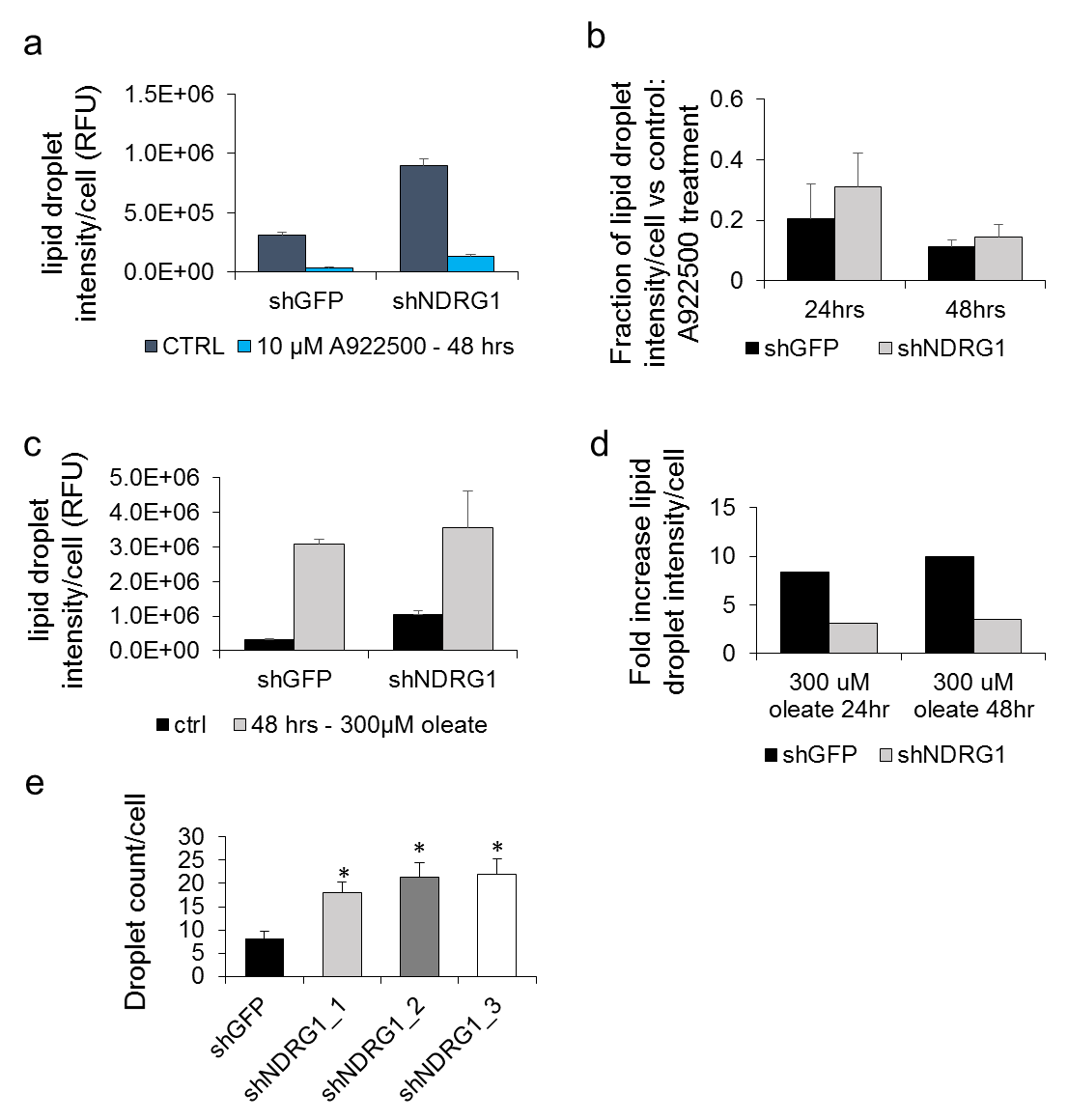
**

**Figure S6. Validation of Lipid Droplet Assays and NDRG1 Related Lipid Storage Biology.** (A,) Stable shNDRG1 and shGFP expressing SKBR3 cells were treated with the diacylglyceride acyl transferase 2 inhibitor A-922500 (10 μM) for the indicated times, fixed, lipid droplets were stained with BODIPY 493/503, nuclei were counterstained with Hoechst, and cells were imaged and nuclei and lipid droplet counts and intensity analyzed by automated image analysis. (B) Fold increase in lipid droplet intensity was computed at 24 and 48 hrs relative to untreated control cells. Cells treated and analyzed as in A. (C,D) Stable shNDRG1 and shGFP expressing SKBR3 cell lines were cultured with excess oleic acid (300 μM oleic acid coupled with 100 μM fatty acid free bovine serum albumin) in DMEM medium and cells were prepared and analyzed as described in A. (E) Related to figure 5B. Lipid droplet counts were quantified in stable shNDRG1 or shGFP expressing Hs578T cells using additional shRNAs to validate the effect with multiple hairpin constructs. N=6 per group, and comparisons were made with Student’s 2-sided T-test. Error bars represent standard deviation, and all bars represent means, or ratios of means, as indicated.


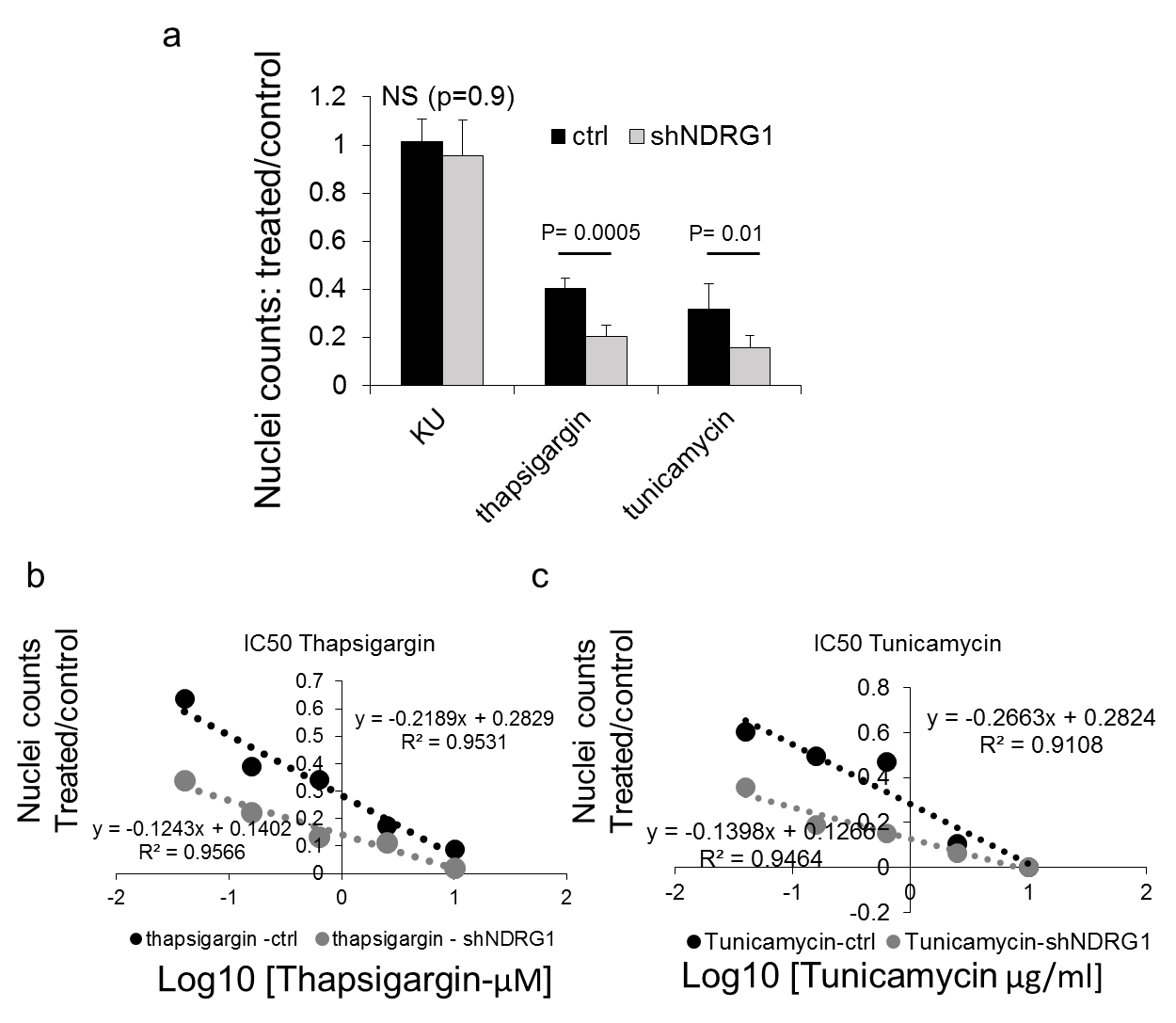


**Figure S7. NDRG1 Silencing Sensitizes SKBR3 Cells to ER Stress.** (A) Nuclei counts of cells treated with the mTOR ATP competitive inhibitor Ku-0063794 (1μM), thapsigargin (1μM), or tunicamycin (5 μg/ml) for 48 hrs (n=3/group, Bars represent mean nuclei counts of treated cells/mean nuclei counts of untreated cells, and error bars are standard deviation). (B,C) related to figures 5 E,F. Lethal Dose 50 was determined for tunicamycin and thapsigargin in stable SKBR3 expressing shNDRG1 or shGFP. N=3/group.

**Additional methods**

**NDRG1 overexpression construct cloning**

The NDRG1 gene was obtained in pDSRED N2 (a gift from Dr. Sushant Krishna Kachhap). A point mutation was corrected using the Q5 site directed mutagenesis kit (New England Biolabs cat# E0554S). The Phusion high fidelity PCR kit was used to amplify the full length gene (encoding amino acids 1- 394) and constructs omitting the intrinsically disordered N and C temini - ΔN amino acids = 33-394, ΔC amino acids = 33-394 (New England Biolabs cat# E0553). Sequences were verified by Sanger sequencing at the SUNY-Albany Center for Functional Genomics Molecular Core Facility. PCR and restriction digest and ligation based subcloning into pMarxIV-hygro was carried out using standard methods.

**Virus production and cell transduction**

HEK293T cells were transfected using X-treme gene HP transfection reagent (Roche) with Gag/pol, Rev, VsVG (Invitrogen Virapower), and shRNA constructs. X-treme gene reagent was used at a ratio of 2:1 (volume: µg) following manufacturer’s protocols. After overnight incubation, transfection medium was replaced by viral production medium - DMEM high glucose, plus glutamine and pyruvate, supplemented with 10% fetal bovine serum and 1% BSA. Virus containing medium was collected daily. Virus containing medium was pooled, centrifuged at 1000 X g, and passed through 0.45 μm filter and applied directly to cell lines for infection, or aliquoted (1ml) and frozen at -80°C. Cells were grown to 70%-80% confluence, transduced for 48 hrs with 1:10 diluted virus in complete DMEM medium containing 8 ug/ml polybrene (Sigma), and cells were selected in medium containing 1 µg/ml puromycin (Sigma).

For retrovirus production, Phoenix AMPHO cells were transfected with Flag-tagged versions of NDRG1 cloned in the MarxIV vector, and the empty MarxIV vector was used as a control. Culture medium was collected and processed as described for lentivirus production above, with the exception that retrovirus pools were not frozen.

**Fluorescence microscopy**

Cells were grown in 96 Well Flat Clear Bottom Black Polystyrene TC-Treated Microplates (Corning catalog # 3603). All Cells were fixed by the addition of 100 µl 5% formaldehyde in PBS for 15 minute at room temperature, followed by three consecutive five minute washes in PBS (200 µl). All cells were co-stained with the DNA dye Hoechst (1 µg/ml).

For lipid droplet analysis, fixed cells were stained with 10 μg/ml BODIPY 493/503 for 30 minutes at room temperature (ThermoFisher D-3922), followed by three consecutive five minute washes in PBS (200 µl). Cells were imaged in PBS using appropriate fluorescence filter sets.

**Determination of shRNA mediated NDRG1 silencing by multiple shRNAs**

At 4-5 days post selection, SKBR3 cells transduced with each shNDRG1 expressing virus or negative controls (empty vector and sh-eGFP) were plated at 10,000 cells per well in 96 well plates. After at least 24 hrs cells were fixed analyzed by anti-NDRG1 immunofluorescence as described above. Images were acquired at 20X magnification on an inverted fluorescence microscope (Olympus IX-81) fitted with a Retiga 6000 CCD Camera using Metamorph software or on the InCell Analyzer 2200 high content microscopy platform (GE Healthcare). All images were acquired using the same exposure time to allow quantitative analysis and comparison of staining intensity.

In order to quantify signal a common phalloidin threshold was determined using ImageJ stack threshold operation. The signal was binarized, and holes were filled using binary operations. The mask was then applied to the target staining image using the region of interest manager, and average signal in cells was obtained. Average NDRG1 signals were compared for cells from at least three wells, and compared using Student’s T-test. Several shRNAs achieved >80% signal reduction using this method in SKBR3 cells. Three effective shRNAs were selected for further use – two targeting protein coding regions of NDRG1 mRNA, and the third targeting the 3’ UTR (TRCN0000084047, TRCN0000084044, TRCN0000084043, GE Healthcare). The GFP and empty vector controls were from Addgene (pLKO.1 GFP shRNA, Plasmid #30323, and pLKO.1 puro Plasmid #8453).
