# Supplementary material for: NDRG1 regulates neutral lipid metabolism in breast cancer cells
Source: Breast Cancer Res. 2018 Jun 14;20:55. doi: 10.1186/s13058-018-0980-4 (PMC6001025; doi:10.1186/s13058-018-0980-4)
Supplement: Supplementary file 2 — Supplemental tables. (DOCX 30 kb) [file 13058_2018_980_MOESM2_ESM.docx]

Additional file 2

Table S1

| Gene | Corr | Gene | Corr | Gene | Corr | Gene | Corr |
| --- | --- | --- | --- | --- | --- | --- | --- |
| *ABAT* | -0.54 | *EVL* | -0.44 | *AGR2* | -0.42 | *ZFYVE16* | -0.41 |
| *NAT1* | -0.53 | *STK36* | -0.44 | *FAM198B* | -0.42 | *CAPN13* | -0.41 |
| *C9ORF116* | -0.51 | *PRR15* | -0.44 | *LRG1* | -0.42 | *ELOVL5* | -0.41 |
| *AR* | -0.5 | *DYX1C1* | -0.44 | *ARSG* | -0.42 | *TPRG1* | -0.41 |
| *FOXA1* | -0.48 | *NOSTRIN* | -0.44 | *DYNLRB2* | -0.42 | *PHYHD1* | -0.41 |
| *GATA3* | -0.48 | *SIDT1* | -0.44 | *CIRBP* | -0.42 | *C1ORF64* | -0.41 |
| *TFF3* | -0.48 | *HSD17B4* | -0.44 | *OGN* | -0.42 | *CYP21A2* | -0.41 |
| *FUT8* | -0.47 | *SLC22A5* | -0.44 | *IFT88* | -0.42 | *DNAJC12* | -0.41 |
| *REEP6* | -0.47 | *FAM214A* | -0.44 | *ACOT4* | -0.42 | *FBP1* | -0.41 |
| *CCDC176* | -0.47 | *NEK10* | -0.44 | *THSD4* | -0.42 | *KIF9* | -0.41 |
| *AGR3* | -0.47 | *SLC44A4* | -0.44 | *GREB1* | -0.42 | *BBS1* | -0.41 |
| *IGFALS* | -0.46 | *TCTN1* | -0.44 | *CCDC96* | -0.42 | *ABCC11* | -0.41 |
| *LRRC46* | -0.46 | *RSPH1* | -0.44 | *KIAA1324* | -0.41 | *EFCC1* | -0.41 |
| *CASC1* | -0.46 | *ZMYND10* | -0.44 | *DACH1* | -0.41 | *ALDH6A1* | -0.41 |
| *DNALI1* | -0.45 | *TMEM25* | -0.43 | *FSIP1* | -0.41 | *PGPEP1* | -0.4 |
| *ABCC8* | -0.45 | *ANXA9* | -0.43 | *MYO5C* | -0.41 | *CA12* | -0.4 |
| *SAMD15* | -0.45 | *ECI2* | -0.43 | *ATP5G2* | -0.41 | *NME3* | -0.4 |
| *CCDC170* | -0.45 | *IFT22* | -0.43 | *ERBB4* | -0.41 | *APH1B* | -0.4 |
| *MLPH* | -0.45 | *ANKRA2* | -0.43 | *CRIP1* | -0.41 | *PYCARD* | -0.4 |
| *LRRC48* | -0.45 | *ANKRD42* | -0.43 | *SLC7A8* | -0.41 | *PTPRT* | -0.4 |
| *ESR1* | -0.44 | *TP53TG1* | -0.43 | *WFS1* | -0.41 | *NINJ1* | -0.4 |
| *IL6ST* | -0.44 | *MYB* | -0.43 | *SPATA4* | -0.41 | *NME5* | -0.4 |
| *ZNF552* | -0.44 | *SUOX* | -0.42 | *TBC1D9* | -0.41 | *ACSM1* | -0.4 |
| *SCUBE2* | -0.44 | *TMEM144* | -0.42 | *RGS22* | -0.41 | *FBXO36* | -0.4 |
| *DNAH7* | -0.44 | *TTC8* | -0.42 | *IGBP1* | -0.41 | *RALGPS2* | -0.4 |

Table S2

| TCGA – NDRG1 positively correlated gene list | | | | | |
| --- | --- | --- | --- | --- | --- |
| Gene | Corr | Gene | Corr | Gene | Corr |
| *AGO2* | 0.52 | *RDH10* | 0.44 | *SBSN* | 0.42 |
| *ADM* | 0.52 | *SLC2A1* | 0.44 | *EN1* | 0.42 |
| *VEGFA* | 0.52 | *PLOD1* | 0.44 | *MATN4* | 0.42 |
| *CA9* | 0.51 | *EGFR* | 0.44 | *IL20RB* | 0.42 |
| *ENO1* | 0.49 | *FANCE* | 0.44 | *AMD1* | 0.42 |
| *BOP1* | 0.47 | *PKP1* | 0.44 | *DLX5* | 0.42 |
| *ATP6V1C2* | 0.46 | *MICALL1* | 0.44 | *IL12RB2* | 0.42 |
| *KRT16* | 0.46 | *PGK1* | 0.44 | *NARF* | 0.42 |
| *LEMD1* | 0.46 | *FOXL1* | 0.43 | *GABBR2* | 0.42 |
| *YEATS2* | 0.46 | *B3GNT5* | 0.43 | *GSDMC* | 0.41 |
| *SLC25A32* | 0.45 | *OPRK1* | 0.43 | *ATAD2* | 0.41 |
| *DSCC1* | 0.45 | *ART3* | 0.43 | *RNF182* | 0.41 |
| *ABTB2* | 0.45 | *SCEL* | 0.43 | *ANKS6* | 0.41 |
| *PSAT1* | 0.45 | *PTDSS1* | 0.43 | *MRPL15* | 0.41 |
| *TMCC2* | 0.45 | *KCNK5* | 0.43 | *MED30* | 0.41 |
| *PDK1* | 0.45 | *CHRM2* | 0.43 | *DLX6* | 0.41 |
| *RGS20* | 0.45 | *FZD9* | 0.43 | *MTHFD1L* | 0.41 |
| *TMEM65* | 0.45 | *HSF1* | 0.43 | *MTBP* | 0.4 |
| *SLC6A2* | 0.45 | *YBX1* | 0.42 | *CHRAC1* | 0.4 |
| *RRAGD* | 0.45 | *GJB6* | 0.42 | *CTPS1* | 0.4 |

Table S3

| Luminal Breast Cancer Protein Expression in NDRG1 Altered Cases | | | |
| --- | --- | --- | --- |
| Protein | fold-change vs NDRG1 unaltered | p-Value | q-Value |
| ESR1 | -3.5 | 1.24E-08 | 4.05E-07 |
| PGR | -2.5 | 2.5E-08 | 6.55E-07 |
| GATA3 | -2 | 3.07E-12 | 4.02E-10 |

Table S4

| Gene expression in NDRG1 Altered cases compared with unaltered cases | | | |
| --- | --- | --- | --- |
| Gene | fold-change:  Altered vs Unaltered | p-Value | q-Value |
| *HORMAD1* | 4.56 | 1.10E-09 | 3.06E-08 |
| *KRT16* | 4.17 | 3.57E-13 | 5.09E-11 |
| *PSAT1* | 3.41 | 5.30E-13 | 6.98E-11 |
| *KRT6A* | 2.91 | 1.09E-08 | 2.01E-07 |
| *KRT6C* | 2.79 | 4.94E-09 | 1.05E-07 |
| *EGFR* | 2.6 | 7.89E-10 | 2.30E-08 |
| *GATA3* | -3.49 | 4.13E-13 | 5.61E-11 |
| *PGR* | -3.53 | 6.01E-13 | 7.61E-11 |
| *FSIP1* | -3.84 | 1.37E-09 | 3.63E-08 |
| *DACH1* | -4 | 1.35E-11 | 7.85E-10 |
| *TFF3* | -4.08 | 5.37E-13 | 7.02E-11 |
| *AR* | -4.16 | 3.39E-14 | 7.49E-12 |
| *GFRA1* | -5.1 | 1.02E-13 | 1.85E-11 |
| *SCUBE2* | -5.1 | 4.98E-16 | 2.68E-13 |
| *NAT1* | -6.25 | 4.93E-18 | 6.53E-15 |
| *AGR2* | -6.6 | 2.24E-12 | 2.07E-10 |
| *FOXA1* | -7.5 | 3.49E-12 | 2.80E-10 |
| *ESR1* | -7.61 | 1.68E-13 | 2.76E-11 |
| *AGR3* | -13.46 | 9.59E-15 | 2.67E-12 |

Table S5

| 42 Gene Pan-Cancer NDRG1 Correlation Signature | | | |
| --- | --- | --- | --- |
| Gene | Ontology | Gene | Ontology |
| *HILPDA* | positive regulation of lipid storage | *SLC2A1* | glucose transport |
| *KCTD11* | protein ubiquitination | *PYGL* | glycogen catabolic process |
| *ERO1L* | cellular response to hypoxia | *ALDOA* | glycolytic process |
| *NDRG1* | cellular response to hypoxia | *ENO1* | glycolytic process |
| *EGLN3* | response to hypoxia | *ENO2* | glycolytic process |
| *GJB2* | response to ischemia | *GAPDH* | glycolytic process |
| *DSC2* | cellular response to starvation | *HK2* | glycolytic process |
| *MXI1* | transcription corepressor activity | *PFKFB4* | glycolytic process |
| *ZNF395* | transcription, DNA-templated | *PFKP* | glycolytic process |
| *NDUFA4L2* | mitochondrial respiratory chain complex IV | *PGAM1* | glycolytic process |
| *ADM* | angiogenesis | *PGK1* | glycolytic process |
| *VEGFA* | angiogenesis | *PKM* | glycolytic process |
| *ESM1* | angiogenesis | *TPI1* | glycolytic process |
| *ITGA5* | angiogenesis | *PDK1* | regulation of glucose metabolic process glycolysis |
| *PGF* | angiogenesis | *LDHA* | carbohydrate metabolic process glycolysis |
| *CA9* | bicarbonate transport | *BNIP3L* | mitochondrial protein catabolic process |
| *PTHLH* | cAMP metabolic process | *BNIP3* | positive regulation of autophagy |
| *PPFIA4* | neurotransmitter secretion | *AK4* | nucleoside triphosphate biosynthetic process |
| *PTK2* | microtubule cytoskeleton organization | *ANKRD37* | nucleus |
| *KRT16* | cytoskeleton | *P4HA1* | oxidation-reduction process |
| *TGFB1* | epithelial to mesenchymal transition | *P4HA2* | oxidation-reduction process |

Table S6

| Cell Line | ER status | HER2 status |
| --- | --- | --- |
| MCF7 | + | - |
| BT474 | + | + |
| Hs578T | - | - |
| MDA-MB-231 | - | - |
| MDA-MB-468 | - | - |
| SKBR3 | - | + |
| HCC1569 | - | + |
